# Advances in laboratory diagnosis of neonatal hyperbilirubinemia and peculiarities in plateau regions: a review of evidence

**DOI:** 10.3389/fped.2026.1782889

**Published:** 2026-04-09

**Authors:** Changqing Tang, Lijun Xia, Dengbo Xu

**Affiliations:** 1 Department of Clinical Laboratory Medicine, Wuhou District People’s Hosptial, Chengdu, China; 2 Department of Clinical Laboratory Medicine, Wuhou District Health Hospital Woman & Children, Chengdu, China

**Keywords:** characteristics, clinical, hyperbilirubinemia, laboratory medicine, management strategies, neonatal, plateau, regions

## Abstract

High-altitude neonatal hyperbilirubinemia refers to a common condition in newborns residing at elevations of 2,500 meters or higher. This condition arises from the combination of high-altitude environmental factors—such as hypoxia and low atmospheric pressure—with physiological characteristics specific to newborns, including immature bilirubin metabolism and increased red blood cell destruction. These factors collectively lead to abnormally elevated serum bilirubin levels. However, studies on the prevalence and associated factors of neonatal hyperbilirubinemia have remained controversial due to influences from cultural, demographic, geographic, climatic, and clinical conditions. The World Health Organization recommends a one-day hospital stay after uncomplicated delivery, jaundice assessment prior to discharge, and screening for hyperbilirubinemia on the third and seventh days after birth. Due to constraints such as the harsh environment of plateau regions, regional economic deprivation, insufficient investment in medical resources, and risks associated with genetic variations among populations, implementing these recommendations proves particularly challenging in China, especially in high-altitude ethnic minority areas. Therefore, medical laboratory technology plays a pivotal role in the early screening, diagnosis, and treatment efficacy assessment of neonatal hyperbilirubinemia in high-altitude regions. This systematic review summarizes recent advances in the epidemiological characteristics, etiological mechanisms, diagnostic strategies, and therapeutic interventions for neonatal hyperbilirubinemia in high-altitude regions. It also examines the application value of rapid detection technologies and specific intervention strategies in plateau areas. Finally, it outlines future research directions, providing evidence-based guidance and practical recommendations for clinical practitioners.

## 1 Introduction

Neonatal hyperbilirubinemia is one of the most common clinical issues in the neonatal period. Most newborns develop jaundice during the first week after birth (1). While the vast majority of cases represent a physiological process, abnormally elevated bilirubin levels or the presence of other pathological factors may lead to acute bilirubin encephalopathy or kernicterus, causing irreversible neurological damage (2). Hyperbilirubinemia in newborns from high-altitude regions exhibits distinct geographical and ethnic specificity. Due to prolonged residence at elevations exceeding 4,000 meters on average, populations in these areas have evolved unique physiological adaptations (3), including polycythemia, elevated hemoglobin concentration, and altered activity of enzymes involved in bilirubin metabolism. Early studies indicated (4) that infants born at 3,100 m elevation had twice the incidence of jaundice compared to those born at 1,600 m.

Given the distinct nature and severity of neonatal hyperbilirubinemia in high-altitude regions, coupled with significant advancements in diagnostic techniques and therapeutic strategies in recent years, this paper comprehensively reviews the clinical characteristics and management approaches of this condition from a laboratory medicine perspective. It aims to provide guidance for clinical practice and future research directions.

## 2 Article types

### 2.1 Epidemiological characteristics of neonatal hyperbilirubinemia in plateau regions

Internationally, neonatal hyperbilirubinemia ranks among the most common neonatal disorders, defined as jaundice resulting from serum bilirubin levels exceeding physiological thresholds (typically ≥171 μmol/L in full-term infants). Core contributing factors include immature neonatal liver metabolism, hemolysis, and infections that cause excessive bilirubin production or excretion disorders. Severe cases may lead to kernicterus, resulting in irreversible neurological damage. Regions like Asia and Africa face higher risks of severe outcomes due to prevalent genetic polymorphisms, whereas developed countries report kernicterus incidence rates of only 1/43,000–1/79,000 live births. In clinical practice, countries like the US and UK have updated guidelines to standardize intervention thresholds and protocols. Europe and America have optimized prevention through measures like anti-D serum prophylaxis and early screening. However, developing countries often face delayed interventions due to scarce medical resources and inadequate screening/follow-up systems. In some regions, delayed hospital admission for affected infants results in higher risks of severe disease and sequelae compared to developed nations. Overall prevention efforts are significantly impacted by unequal resource distribution.

### 2.2 Incidence and distribution of neonatal hyperbilirubinemia in plateau regions

In China, the disease burden of neonatal hyperbilirubinemia is significantly higher in the Tibet region than in low-altitude areas. Studies indicate that among newborns in Lhasa (elevation 3,658 m), the incidence of hyperbilirubinemia is elevated, with markedly increased proportions of severe hyperbilirubinemia (TSB 342–<427 μmol/L) and extremely severe hyperbilirubinemia (TSB 427–<510 μmol/L). A retrospective study of 299 high-altitude newborns with hyperbilirubinemia (5) revealed a peak TSB of 342.5 ± 65.23 μmol/L (range 255.1–538.9), with severe hyperbilirubinemia affecting 36.5% of cases. Acute kernicterus occurs in approximately 5.5% of cases of severe hyperbilirubinemia. These figures substantially exceed reports from lowland areas, highlighting the unique challenges and risks associated with neonatal bilirubin metabolism at high altitudes. Prior research (6) has similarly demonstrated elevated bilirubin levels and a higher incidence of hyperbilirubinemia in high-altitude newborns compared to those in lowland regions.

### 2.3 Analysis of risk factors for neonatal hyperbilirubinemia in plateau regions

We found significant variations across national guidelines regarding risk factors for neonatal hyperbilirubinemia. Only preterm birth and hemolytic disease are explicitly listed as universally recognised risk factors in all guidelines (7). The criteria for other potential risk factors vary among national guidelines. Additionally, an analysis of guidelines from 12 countries revealed that nearly all guidelines identify preterm birth, exclusive breastfeeding, and G6PD deficiency as risk factors (8). Some guidelines also include intracranial hematoma or bruising and male gender as risk factors. However, the NICE guideline notes that there is insufficient evidence, with most studies showing no significant association between these factors and hyperbilirubinemia.

Compared to guidelines established by the WHO, World Health Organisation, Chinese Medical Association, and national health organisations, risk factors for neonatal hyperbilirubinemia in high-altitude regions include not only common characteristics such as preterm birth, low birth weight, and perinatal asphyxia (5), but also other specific factors. Table 1 illustrates the unique risk factors for neonatal hyperbilirubinaemia in high-altitude regions.

**Table 1:** Risk factors and mechanisms of neonatal hyperbilirubinemia in high-altitude regions.

Risk factors	Specific factors	Pathogenesis	References
Physiological factors	High-altitude environments ethnic characteristics	Increased red blood cell production elevated bilirubin synthesis	Li C, Li X (9)
			Asas-Jinde (10)
Infectious factors	TORCH infections Sepsis	Accelerated red blood cell destruction hepatic dysfunction	Ren X (11)
Hemolytic factors	ABO hemolysis irregular antibodies	Immune hemolysis excessive bilirubin production	JACKSON (12)
			Mandal (13)
Perinatal factors	Premature placental aging Asphyxia obstructed labor	Hypoxia Acidosis Hepatic injury	Grant ID (14)
			Ali KZ (15)
Feeding factors	Delayed lactation onset inadequate feeding	Enhanced enterohepatic circulation	Sato H (16)
			Niermeyer (17)

#### 2.3.1 Infectious factors

TORCH infection rates vary across regions due to differences in local economy, culture, and environment. Generally, high-altitude areas exhibit higher rates owing to limited transportation access, suboptimal healthcare infrastructure, inadequate medical equipment and testing capabilities, insufficient public awareness of TORCH infections, and relatively weak hygiene knowledge and self-care practices (9). The northwest plateau region exhibits elevated TORCH infection rates, with Toxoplasma gondii positivity reaching 3.4%, CMV infection at 2.81%, and HSV infection at 15.82%—all surpassing rates in other regions (10). These infections heighten the risk of neonatal hyperbilirubinemia.

#### 2.3.2 Hemolytic factors

Due to the hypoxic environment at high altitudes, residents in high-altitude regions exhibit significantly higher levels of RBC, HGB, and PLT compared to those in low-altitude areas of China (11). Additionally, beyond ABO and Rh hemolysis, hemolysis caused by irregular antibodies is relatively common among high-altitude populations (12). Studies report (13) a high detection rate of irregular antibodies in populations residing at high altitudes in northern India, posing a serious threat to the health and lives of pregnant women and newborns.

#### 2.3.3 Breastfeeding issues

Studies have identified inadequate breastfeeding as an independent risk factor for neonatal hyperbilirubinemia in breastfed infants (14). Insufficient feeding may increase intestinal bilirubin absorption due to energy deficiency, thereby impairing hepatic bilirubin conjugation and excretion, ultimately leading to neonatal hyperbilirubinemia. Traditional beliefs contribute to feeding misconceptions in Tibet, such as delayed initiation of breastfeeding after birth, inadequate environmental hygiene, and even failure to provide colostrum (15, 16). These practices result in neonatal energy deficiency, delayed meconium passage, and increased enterohepatic circulation.

#### 2.3.4 Environmental factors

Significant diurnal temperature fluctuations at high altitudes predispose newborns to cold injury syndrome (17), impairing peripheral circulation and reducing hepatic blood and oxygen supply, which further inhibits bilirubin metabolism (18).

#### 2.3.5 Perinatal factors

Preterm Birth/Low Birth Weight: Placental maturity in pregnant women at high altitudes occurs earlier than in lowland regions (19). While placental maturity reaches Grade III around 38 weeks in lowland pregnancies, it advances to approximately 32 weeks in high-altitude pregnancies due to hypoxia (20). This accelerated placental ageing may increase preterm birth risk. Preterm infants (<37 weeks of gestation) exhibit poorer liver development, with UGT1A1 activity only one-third that of term infants. Under the combined effects of high-altitude hypoxia, their metabolic capacity nearly “collapses,” resulting in hyperbilirubinemia incidence 3–4 times higher than in term infants (21).

In summary, neonatal hyperbilirubinemia in high-altitude regions results from multiple factors: hypoxia as the primary driver, combined with physiological immaturity, exacerbated by genetic predisposition and clinical complications. Clinical management requires targeted interventions, such as early oxygen therapy, bilirubin monitoring, infection prevention, and ensuring adequate nutrition, to reduce the risk of severe complications.

### 2.4 Pathophysiology of neonatal hyperbilirubinemia

#### 2.4.1 Physiological characteristics of neonatal bilirubin metabolism in plateau regions

##### 2.4.1.1 Congenital immaturity of liver function

**Figure 1:**
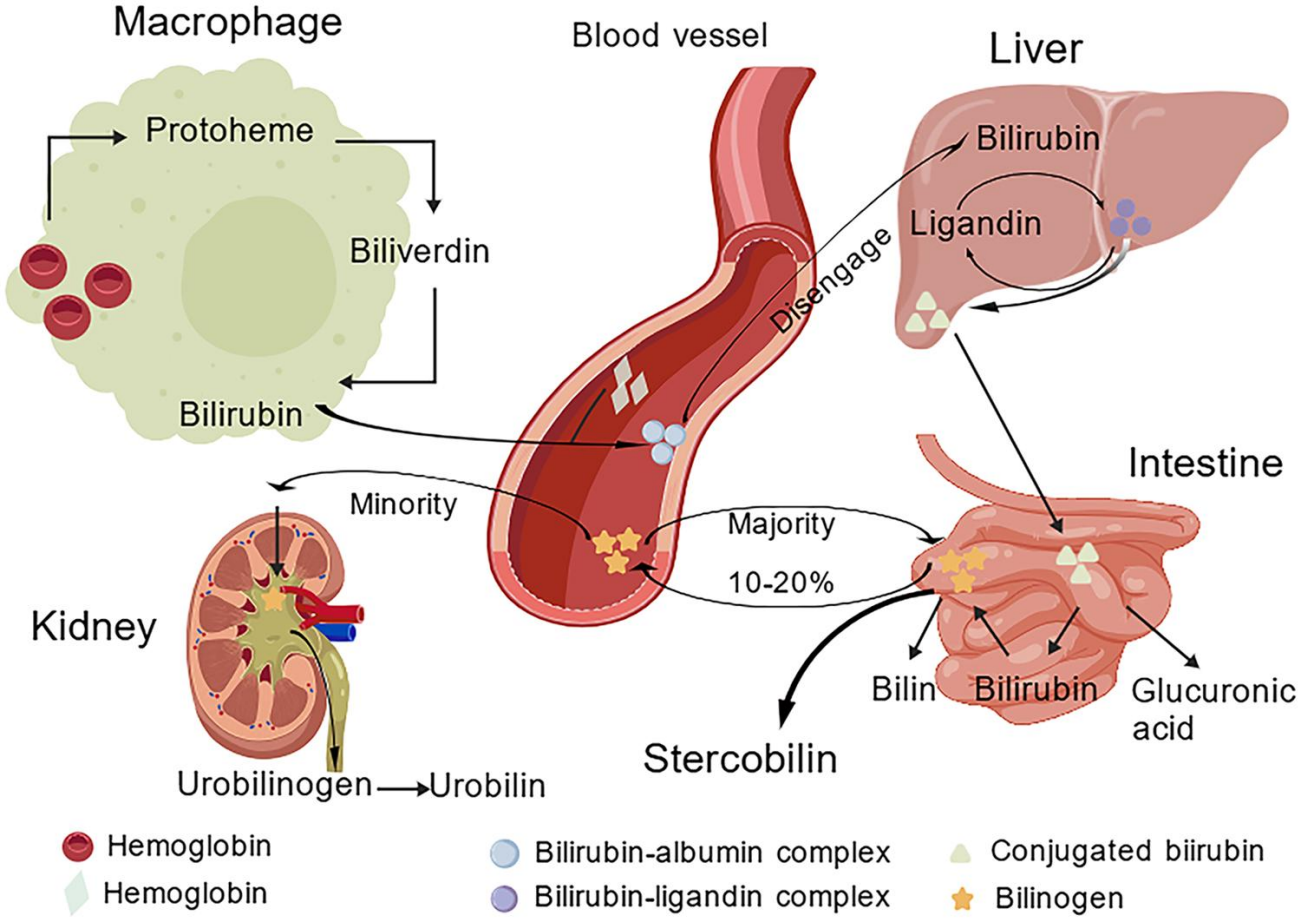
Diagram illustrating bilirubin metabolism, showing hemoglobin breakdown in macrophages, transport of bilirubin in the blood, uptake and processing by the liver, conversion in the intestine to bilirubin and stercobilin, and elimination via the kidney as urobilin. Various chemical forms and complex transitions are depicted for each organ. Color-coded key denotes hemoglobin, bilirubin complexes, conjugated bilirubin, bilinogen, and related molecules.

In addition to low UGT1A1 activity, insufficient levels of Y protein and Z protein (carrier proteins responsible for uptake of blood-derived indirect bilirubin) within neonatal hepatocytes hinder the entry of indirect bilirubin into hepatocytes for metabolism (22); Concurrently, elevated intrahepatic cholestasis risk (due to immature bile duct development) impedes direct bilirubin excretion, further exacerbating jaundice (23).The process by which bilirubin enters the bloodstream and is transported to the liver (Figure 1).

##### 2.4.1.2 Compensatory activation of the erythrocytic system

Neonates inherently undergo “physiological hemolysis” after birth (replacement of fetal haemoglobin with adult haemoglobin) (24), a process exacerbated by high-altitude hypoxia. Studies reveal that in high-altitude regions, neonatal red blood cell variability increases by 27%, hematocrit rises by 3%, and haemoglobin levels climb by 0.4 g (25). These changes stem from increased renal erythropoietin secretion, which stimulates the bone marrow to produce more red blood cells while accelerating the destruction of old red blood cells. This dual effect leads to an increased supply of the precursor to bilirubin (haemoglobin).

#### 2.4.2 Effects of high-altitude environments on neonatal liver function

##### 2.4.2.1 Accelerated bilirubin production

High-altitude hypoxia (where the partial pressure of oxygen significantly decreases at elevations ≥3,000 meters) stimulates compensatory erythropoiesis in newborns (25). This concurrently shortens the lifespan of red blood cells (due to reduced membrane stability and increased fragility in hypoxic conditions). The massive release of haemoglobin is converted into unconjugated bilirubin (indirect bilirubin), overwhelming the liver's metabolic capacity. For instance, research indicates (26) that the rate of neonatal red blood cell destruction at 3,100 meters elevation is 20%–30% higher than at 1,600 meters, leading to increased indirect bilirubin production and consequently elevated serum bilirubin levels.

Concurrently, studies on hyperbilirubinemic newborns indicate these infants experience oxidative stress, with serum antioxidant enzyme activity decreasing as bilirubin levels rise (27). Furthermore, the insufficient oxygen-carrying capacity of red blood cells stimulates haemoglobin production to ensure oxygen supply, thereby increasing unconjugated bilirubin in the blood.

##### 2.4.2.2 Inhibition of liver enzymes involved in bilirubin metabolism

The neonatal liver is inherently underdeveloped, with hepatic uridine diphosphate glucuronosyltransferase (UGT1A1) activity reaching only 1%–2% of adult levels (in term infants). High-altitude hypoxia significantly elevates aryl hydrocarbon receptor (AhR) expression (28), whose receptors, PXR and CAR, can further regulate and inhibit UGT1A1 activity. UGT1A1 is the key enzyme converting indirect bilirubin into direct bilirubin (excretable via bile). Its reduced activity directly causes “blockage” in indirect bilirubin metabolism, leading to its accumulation in the bloodstream (29).

Concurrently, research indicates that hypoxia-induced liver injury at high altitudes may promote direct bilirubin entry into the bloodstream. Subjects’ red blood cells may be unable to carry sufficient oxygen, making them more susceptible to hypoxia-induced liver damage. Both *in vivo* and *in vitro* experiments demonstrate that bilirubin exhibits antioxidant properties (30). Consequently, the hypoxic environment at high altitudes triggers oxidative stress and apoptosis, leading to liver damage and impaired bilirubin metabolism (31).

##### 2.4.2.3 Reduced hepatic glycogen reserves and diminished liver metabolic capacity

Hypoxia inhibits glycogen synthesis in human hepatocytes, leading to insufficient hepatic glycogen reserves (32). Liver metabolism of bilirubin requires energy expenditure. Reduced glycogen reserves further impair hepatocyte uptake and conjugation capacity for bilirubin, creating a vicious cycle of “insufficient metabolic capacity-bilirubin accumulation.” As early as 1988, animal studies (simulating hypoxia at 8,000 meters altitude) demonstrated that neonatal rat hepatocytes exhibited over 40% lower glycogen content compared to low-altitude controls, accompanied by significantly reduced bilirubin binding capacity (33).

### 2.5 Genetic factors in neonatal hyperbilirubinemia at high altitude

Genetic variations among different ethnic groups/populations result in differing adaptability to high-altitude hypoxia and varying efficiencies in bilirubin metabolism among newborns. Among these, UGT1A1 gene polymorphisms represent the most clearly established genetic factor:

#### 2.5.1 UGT1A1 gene variation

**Figure 2:**
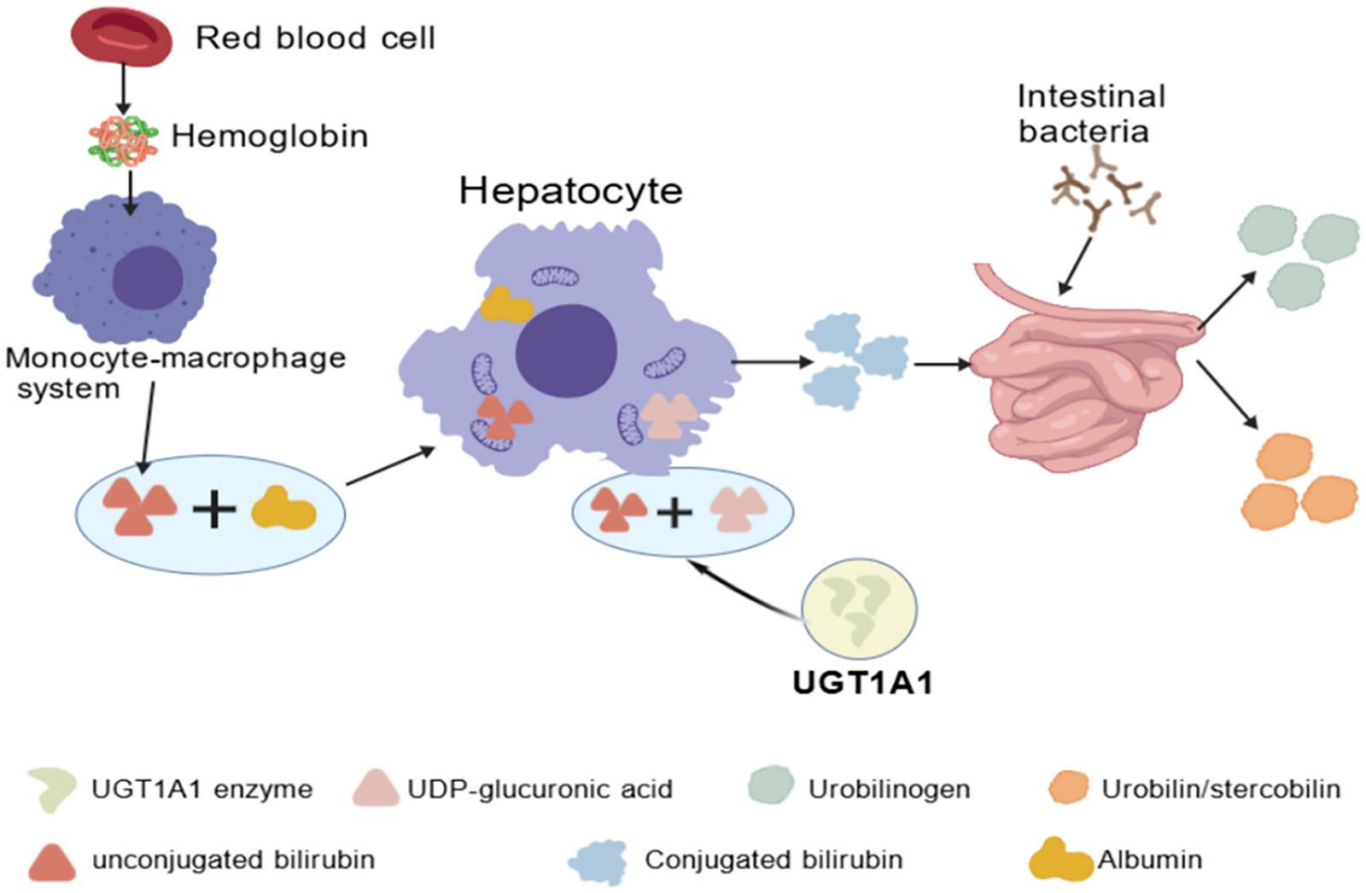
Diagram illustrating the mechanism of UGT1A1 Enzyme in Bilirubin Metabolism. The breakdown of hemoglobin from red blood cells by the monocyte-macrophage system produces unconjugated bilirubin (bound to albumin). This is taken up by hepatocytes, conjugated with UDP-glucuronic acid via UGT1A1 to form conjugated bilirubin, which is excreted into the intestine. Intestinal bacteria convert conjugated bilirubin to urobilinogen; subsequent metabolism generates urobilin/stercobilin, with a portion undergoing enterohepatic circulation. Legend explains symbols for each molecule and enzyme.

Uridine diphosphate glucuronosyltransferase is a key rate-limiting enzyme in bilirubin metabolism (34) (Figure 2). Polymorphisms in the UGT1A1 gene encoding this enzyme may influence bilirubin metabolism enzyme activity (35, 36). As a member of the microsomal membrane UGT1 family, it plays a crucial role in converting toxic bilirubin into a non-toxic form (37). This process, when impaired, leads to the accumulation of unconjugated bilirubin and increased risk of hyperbilirubinemia, a finding validated in conditions such as neonatal jaundice. In studies of unexplained neonatal hyperbilirubinemia (38), research has predominantly focused on the association between UGT1A1 gene polymorphisms and neonatal hyperbilirubinemia. Related studies indicate that the frequency of the A allele at the rs4148323 locus in the UGT1A1 gene correlates with the incidence of severe hyperbilirubinemia (36). Studies in high-altitude populations (39) found that the frequency of the UGT1A1 gene c.211G > A variant (approximately 35%–40%) was significantly higher among Tibetan newborns in plateau regions like Qinghai and Tibet than among Han Chinese newborns in lowland areas (approximately 15%–20%). The incidence of hyperbilirubinemia among newborns carrying this variant was 2.5–3 times higher than that of the wild-type, and they were more prone to developing severe jaundice.

#### 2.5.2 Plateau adaptation genes

Tibetan populations living long-term at high altitudes have developed unique mechanisms for adapting to hypoxic environments. Genetic variations in the hypoxia-inducible factor (HIF) pathway gene endothelial PAS domain-containing protein 1 (EPAS1) are closely associated with hypoxic adaptation (40). Carriers of the “hypoxia adaptation gene” EPAS1 exhibit reduced excessive destruction of red blood cells under hypoxic conditions (41), resulting in relatively lower neonatal bilirubin production. In contrast, Han Chinese newborns migrating to high-altitude regions, lacking such genetic adaptations, face a significantly higher risk of hyperbilirubinemia.

### 2.6 Treatment strategies for neonatal hyperbilirubinemia in plateau regions

#### 2.6.1 Application of phototherapy in neonatal hyperbilirubinemia in plateau regions

**Figure 3:**
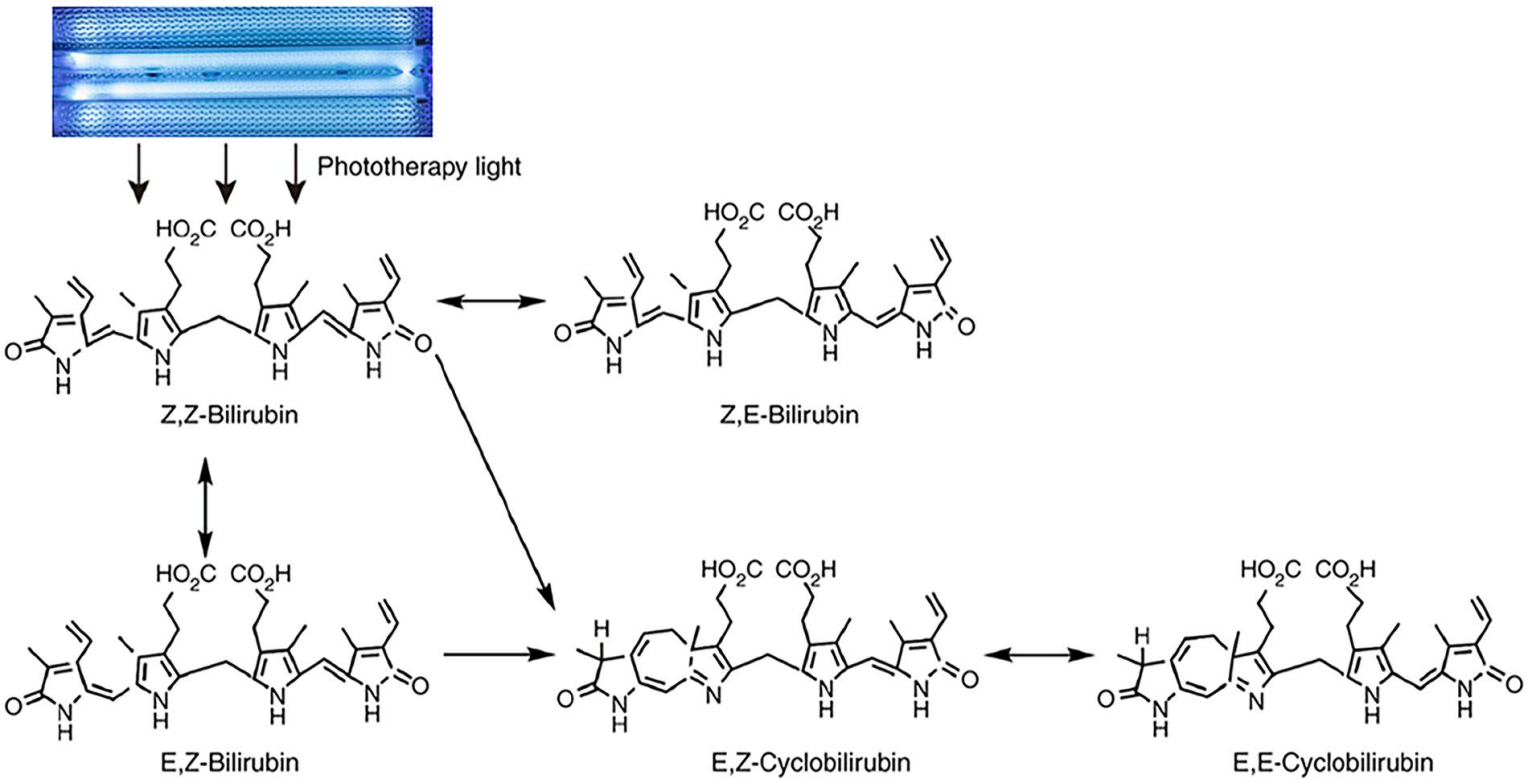
Diagram shows the mechanism of action of phototherapy for neonatal hyperbilirubinemia. Upon exposure to light, non-polar unconjugated bilirubin (Z,Z-bilirubin) in the skin is converted into water-soluble bilirubin isomers, including Z,E-bilirubin, E,Z-bilirubin, E,E-bilirubin, E,Z-cyclobilirubin and E,E-cyclobilirubin.

In the mid-20th century, phototherapy was formally introduced into clinical practice. In 1958, Cremer et al. first reported the effects of phototherapy on bilirubin levels. Subsequently, scientists explored the efficacy and safety of various light sources, including halogen lamps, fibre-optic pads, and blue light (42). Later, blue light irradiation became a standard clinical treatment for severe neonatal hyperbilirubinemia. It controls jaundice progression by altering serum bilirubin (primarily indirect bilirubin) into isomers that are excreted through urine and faeces (43) (Figure 3). Current research indicates that blue light therapy at varying intensities or intermittent continuous phototherapy demonstrates positive effects in reducing neonatal hyperbilirubinemia (44). However, given potential adverse reactions such as hemolysis, allergic reactions, DNA damage, and carcinogenic risks associated with phototherapy, standardised, rational, and regulated blue light therapy protocols should be adopted during treatment.

Research on standalone phototherapy in high-altitude regions is scarce. Some studies have employed combined phototherapy and medication for neonatal hyperbilirubinemia, demonstrating good efficacy in reducing serum bilirubin levels without significantly increasing adverse reactions (45). However, in the unique environment of high altitudes, several special factors must be considered. First, the thin air and high ultraviolet intensity at high altitudes may affect the efficacy and safety of phototherapy. Second, newborns at high altitudes may have higher melanin content in their skin, which can affect the penetration of phototherapy. Third, temperatures are lower at high altitudes, so attention must be paid to maintaining warmth during phototherapy to prevent hypothermia.

#### 2.6.2 Efficacy of pharmacological treatment for neonatal hyperbilirubinemia in plateau regions

As early as 1993, researchers proposed and confirmed that intravenous immunoglobulin (IVIG) could be used to treat hyperbilirubinemia caused by hemolysis (46), particularly in cases of Rh or ABO hemolytic disease. The dosage is 1 g/kg, with repeat administration possible if necessary. Additionally, albumin infusion (1 g/kg) may be administered to infants with hypoalbuminemia to enhance bilirubin binding to albumin and reduce unconjugated bilirubin (47).

Bilirubin production originates from heme catabolism. During the neonatal period, heme released from the breakdown of fetal red blood cells strongly induces heme oxygenase expression, leading to elevated serum bilirubin levels and hyperbilirubinemia (48). In recent years, heme oxygenase inhibitors such as stannous protoporphyrin and stannous mesoporphyrin have demonstrated potential as targeted therapies for hyperbilirubinemia. These agents reduce bilirubin production by inhibiting heme oxygenase (49), thereby offering new avenues for the prevention and treatment of hyperbilirubinemia.

#### 2.6.3 Application of exchange transfusion therapy for neonatal hyperbilirubinemia in plateau regions

Exchange transfusion therapy originated in the late 1840s, initially performed via open central umbilical venous catheters (50). For newborns with high-risk hyperbilirubinemia (TSB ≥ 510 μmol/L), exchange transfusion is a critical life-saving intervention (51). It rapidly reduces serum bilirubin levels, alleviates neonatal hemolytic symptoms, and prevents neonatal bilirubin encephalopathy (48). During an exchange transfusion, it is essential to select appropriate blood products, preferably those that match the mother's blood type, to minimise further hemolysis (52). In high-altitude regions, where blood resources are relatively scarce, establishing a comprehensive blood supply system is critical.

#### 2.6.4 Comprehensive management protocol for neonatal hyperbilirubinemia in plateau regions

Treatment of neonatal hyperbilirubinemia should adopt a tiered management strategy based on a comprehensive assessment of bilirubin levels, gestational age at birth, and risk factors. The Guidelines for Diagnosis and Treatment of Neonatal Hyperbilirubinemia (2025) (53) recommend that all newborns undergo visual inspection for skin jaundice at least every 12 h under natural light or white light. Newborns exhibiting visible skin jaundice within 24 h of birth should undergo measurement of TcB or TSB levels, with TSB serving as the primary indicator for risk assessment and intervention guidance.

Phototherapy remains the first-line treatment for hyperbilirubinemia. Recent clinical studies recommend LED light sources with wavelengths between 460 and 490 nm (54). However, due to specific conditions in high-altitude regions—such as environmental factors and ethnic lifestyles—issues like gastrointestinal inflammation and suboptimal treatment outcomes during phototherapy necessitate combined drug regimens. Examples include Yinzhi Huang combined with blue light therapy (45). For extremely severe hyperbilirubinemia, particularly when exchange transfusion criteria are met, prompt exchange transfusion should be performed.

Therefore, special management strategies are required for newborns in high-altitude regions, an imperative dictated by both the physiological fragility of infants and the unique characteristics of the plateau environment. Fundamentally, this population exhibits physiological traits such as immature hepatic metabolism. When compounded by environmental factors like plateau hypoxia, the risk of abnormal bilirubin metabolism increases significantly. Furthermore, local disparities in medical resources and logistical challenges in patient transfer render conventional management inadequate for addressing the urgent diagnostic and therapeutic demands of acute hyperbilirubinaemia. Management centres on “precise prevention and control, rapid intervention, and high-altitude adaptation”. Firstly, establish a tiered screening mechanism utilising high-altitude-adapted detection technologies to conduct early, precise screening of newborns, particularly those at high risk due to oxygen deprivation. Secondly, optimise intervention thresholds by appropriately lowering hyperbilirubinaemia intervention thresholds in light of high-altitude hypoxia's impact on bilirubin metabolism, shortening monitoring intervals to reduce neurotoxicity risks. Thirdly, enhance transport and coordination mechanisms by establishing a closed-loop management system for remote plateau regions: “primary-level screening—bedside intervention—critical care transfer” to minimise transport delays. Fourthly, strengthen environmental adaptation management by considering plateau-specific conditions in instrument maintenance and intervention implementation to ensure feasibility. This specialised management strategy does not negate conventional approaches but optimises them for high-altitude clinical realities. It reduces risks of neonatal bilirubin-related adverse outcomes while aligning with plateau healthcare resource constraints, providing targeted support for this population's health protection.

### 2.7 Contributions and prospects of laboratory medicine in neonatal hyperbilirubinemia at high altitudes

#### 2.7.1 Application of laboratory medicine in diagnosing neonatal hyperbilirubinemia

##### 2.7.1.1 Detection and assessment indicators for neonatal hyperbilirubinemia

The 2022 American Academy of Paediatrics guidelines (55) recommend universal bilirubin screening for newborns before hospital discharge to reduce the incidence of severe hyperbilirubinemia, acute bilirubin encephalopathy, and kernicterus. Consequently, an increasing number of diagnostic indicators have been developed for clinical assessment of NHB severity. Current diagnostic indicators primarily fall into the following categories (Table 2). Photometric transcutaneous bilirubin (TCB) (56), total serum bilirubin(TSB) (57), Carboxyhaemoglobin (58, 59), End-tidal carbon monoxide (60, 61), and Bilirubin-to-albumin (62, 63) ratio each possess their respective advantages and disadvantages.

**Table 2:** Laboratory indicators of hyperbilirubinemia.

Inspection Criteria	Advantages and Disadvantages	References
TCB	Rapid, non-invasive, suitable for dynamic monitoring, but affected by skin tone	Boden B (56)
TSB	Reference standard of diagnostic testing, invasive and does not provide immediate results	Ngashangva (57)
Carboxyhemoglobin COHb	Predicts severe NHB, especially hemolytic-induced risk, requires venous blood	Bailey (58)
		Schutzman (59)
End-tidal carbon monoxide ETCO₂	Offers non-invasive, immediate, low-cost, point-of-care advantages for severe NHB prediction, but lacks reference ranges	Lozar Krivec (60)
		Bhutani (61)
Bilirubin-to-albumin ratio, B/A	Serves as a predictor for severe NHB and acute bilirubin encephalopathy, aids intervention decision-making, requires venous blood	Aasam (62)
		Jiang S (63)

Although an increasing number of diagnostic indicators are available to assess the risk level of NHB disease in clinical evaluations, serum direct bilirubin testing remains the primary indicator for evaluating its severity (64). Any non-invasive bilirubin test results must be validated through invasive diagnostic methods.

##### 4.7.1.2 Serum bilirubin testing methods

Different serum bilirubin detection methods offer distinct advantages based on varying principles. The diazotisation method, which generates azo pigments (absorption peak at 530 nm) through the reaction of bilirubin with diazotised sulfonic acid, is recommended by the American Society for Clinical Pathology as the preferred method for measuring total bilirubin. The vanadate oxidase method employs vanadate as an oxidising agent to convert bilirubin into biliverdin. Due to its simplicity, rapidity, and strong resistance to interference, it has emerged as a superior alternative to the diazonium method (65). Currently, electronic detection instruments based on this principle have been developed for skin photometry (66). High-performance liquid chromatography (HPLC) is widely used for its high sensitivity. Zelenka et al. established a sensitive method for measuring unconjugated and total bilirubin (67). The combination of HPLC with ultra-sensitive thermal lens spectroscopy (TLS) enables the direct detection of serum-free bilirubin (68). Capillary electrophoresis (CE), characterised by efficient separation and minimal sample requirements, is suitable for neonatal bilirubin monitoring (69). Its integration with capillary analysis (FA) enables the determination of free bilirubin and albumin binding capacity (70), providing a basis for assessing the risk of neonatal bilirubin neurotoxicity. Microfluidic chip-based CE/FA technology further meets the demands of point-of-care testing (71). Among electrochemical methods, amperometry is widely applied, encompassing three categories: direct electrode oxidation, molecularly imprinted polymers, and enzyme-catalysed biosensors. For instance, a zirconium-coated silica nanoparticle composite electrode achieved a detection limit of 0.1 nM (72)or even lower. In spectroscopic methods, fluorescence spectroscopy utilises the fluorescence characteristics of the bilirubin-albumin complex at *λ*520 nm to develop a fully automated near-field fluorescence whole blood testing technology (73). In spectrophotometry, direct methods detect red azobenzene pyridine at 454 and 540 nm, suitable for newborns under 2–3 weeks of age (74). Chemiluminescence technology offers high sensitivity advantages, as bilirubin emits light through the peroxyoxalate reaction in organic solvents (75). Breakthroughs have been achieved by integrating multifunctional nanomaterials with the bilirubin-induced fluorescent protein UnaG in bioluminescent sensors (57). Additionally, molecular imprinting technology enables highly selective detection by synthesising polymers with specific cavities, known as molecularly imprinted polymers (MIPs). Fluorescently imprinted polymers can specifically bind *α*-bilirubin through spectral changes (76). In piezoelectric technology, the integration of QCM with MIPs constructed a highly sensitive and selective bilirubin assay system (77). Point-of-care testing devices utilise microfluidics and MEMS technologies for miniaturisation, providing portability, low cost, and minimal sample requirements (78). However, their accuracy is influenced by skin colour, thickness, and measurement site (79). The Table 3 illustrates and analyses several different serological testing methods in terms of accuracy, cost, portability, and suitability for high-altitude conditions.

**Table 3:** Multidimensional comparison of different serum bilirubin assay methods.

Type	Accuracy	Cost	Portability	Environmental (Low Temperature and Low Humidity)	Clinical value in plateau regions
Vanadase method (skin photoometry)	High Approaching the gold standard	Moderate With lower instrument maintenance costs	High Portable electrnic devices,no complex sample preparation required	Strong Withstanding low-temperature and low-humidity environments, good instrument stability	Suitable for primary healthcare settings and rapid on-site screening,reducing sample transport time
Microfludic chip CE/FA technoligy	High Capable of precisely measuring free bilirubin and albumin-binding capacity	Moderately high With chip costs being reasonable; investment remains manageable following instrument miniaturisation	High Compact bedside device requiring minimal sample volume	Strong Miniaturised design minimises the impact of environmental factors on detection, adapting to high-altitude field conditions.	Particularly suitable for neonatal bilirubin testing, enabling assessment of neurotoxicity risk without the need for transfer to a central laboratory.
Point-of-Care Testing (POCT) Devices (Based on Microfluidics/MEMS Technology)	Moderate Influenced by skin conditions (colour, thickness) and measurement location.	Low Low per-test cost, minimal equipment investment threshold.	Extremely high Handheld design requiring no specialised laboratory environment and simple operation	Strong Adaptable to high-altitude, low-temperature, low-humidity environments without complex temperature control equipment.	Most suitable for remote plateau regions, wilderness rescue scenarios, enabling rapid on-site testing and enhancing diagnostic efficiency.
Fully Automated Point-of-Care Fluorescence Whole Blood Testing Technology (Fluorescence Spectroscopy Method)	High With strong fluorescence specificity and good resistance to interference	Moderate High instrument automation and moderate maintenance costs	Moderate to high Compact fully automated equipment, portable and suitable for primary healthcare facilities	Moderate Requires ensuring the stability of the instrument's fluorescence detection module in low-temperature environments	Suitable for primary hospitals in high-altitude regions, reducing sample processing steps, minimising human error, and enhancing testing efficiency

In high-altitude regions, serum bilirubin testing prioritises portable, environmentally adaptable point-of-care technologies. Point-of-care testing (POCT) devices prove most suitable for remote plateau settings, whilst microfluidic chip-based CE/FA techniques better serve precise neonatal screening. Vanadase-based skin photometers balance rapid screening with accuracy. These three approaches may be flexibly selected according to healthcare facility tiers and testing requirements in plateau regions.

#### 2.7.2 Methods for detecting neonatal bilirubin levels in plateau regions

Neonatal bilirubin testing in plateau regions primarily relies on two methods: noninvasive transcutaneous testing and invasive serum testing, both of which require consideration of the unique characteristics of high-altitude environments (80). Noninvasive transcutaneous testing is the preferred screening and monitoring tool, offering painless, convenient, and repeatable advantages, making it suitable for dynamic tracking of neonatal jaundice (81). However, in plateau regions, hypoxia-induced erythropoiesis results in a higher haemoglobin concentration in newborns compared to lowland infants. This alters light scattering and absorption within the skin, potentially causing transcutaneous monitors to underestimate serum bilirubin levels by slight margins, which poses a risk of underestimation (82). Therefore, transcutaneous testing cannot fully replace serum testing, and devices require regular calibration using local serum samples.

Invasive serum testing remains the gold standard for diagnostic purposes. Precise quantification via venous or heel stick blood provides reliable results, distinguishes bilirubin types, and is crucial for identifying aetiology (53). In high-altitude settings, indications for application should be more proactive: immediate testing is required when transcutaneous measurements approach high-risk thresholds, infants exhibit severe jaundice early on, or pathological factors such as hemolysis or infection are suspected. This ensures the accuracy of data to inform treatment decisions (80). Both methods complement each other, forming the foundational network for neonatal bilirubin monitoring in high-altitude regions.

#### 2.7.3 Application of new technologies

With the advancement and innovation of modern medical technology, novel diagnostic methods continue to emerge and gain widespread application. These emerging techniques enhance diagnostic accuracy and efficiency, providing more diverse, scientific, and targeted solutions and pathways for the prevention, screening, and clinical management of neonatal hyperbilirubinemia in high-altitude regions.

##### 2.7.3.1 Artificial intelligence-assisted diagnosis

Leveraging big data analysis and advanced machine learning algorithms, a hyperbilirubinemia risk prediction model specifically designed for newborns in high-altitude regions has been developed (55). By integrating clinical characteristics, environmental factors, and dynamic monitoring data, this model enables precise disease risk assessment and early warning. Simultaneously, the system generates personalised intervention plans based on individual variations, allowing healthcare providers to implement timely and targeted measures that effectively reduce the incidence of severe cases and long-term health impacts (83).

##### 2.7.3.2 Telemedicine technology

Leveraging portable mobile medical devices and high-speed remote data transmission technology, a health management model seamlessly integrates home monitoring with professional hospital guidance (84). Parents can perform daily measurements of critical neonatal physiological indicators at home, with the data uploaded in real-time to medical platforms. Specialised physicians monitor, analyse, and diagnose via remote systems, promptly providing adjustment recommendations. This significantly alleviates the challenges of accessing medical care faced by residents in high-altitude regions due to transportation difficulties and scarce medical resources, enhancing the accessibility and quality of neonatal health management (85).

##### 2.7.3.3 Genetic testing technology

By detecting genetic polymorphisms related to bilirubin metabolism—such as common variant sites in the UGT1A1 gene—this approach identifies newborn cohorts at genetic risk for hyperbilirubinemia. This technology enables early identification of high-risk individuals before clinical symptoms manifest, leveraging the significantly higher sensitivity and specificity of early genetic screening compared to traditional biochemical testing, thereby providing a basis for precision prevention (86). Combined with genetic counselling and early health management strategies, it substantially enhances the targeting of interventions and reduces disease risk.

##### 2.7.3.4 Novel sensing technologies

An ultrasensitive non-enzymatic bilirubin electrochemical sensor (87) employs electrodes modified with polyvinylpyrrolidone-functionalized single-walled carbon nanotubes. Leveraging the high specific surface area and superior electron transport properties of nanomaterials, this approach achieves highly selective and sensitive detection of bilirubin molecules. Concurrently, research (88) demonstrated the technical advantages of non-enzymatic electrochemical sensors for detecting free bilirubin using ceria nanocubes as sensing materials, including rapid detection, low detection limits, and high selectivity. These novel material technologies, when optimised, are suitable for rapid bedside or point-of-care testing in complex environments such as high altitudes, offering reliable new methods for bilirubin level monitoring.

It is well-known that systems for bilirubin detection and monitoring in severe neonatal jaundice remain underdeveloped in low- and middle-income countries. Therefore, introducing affordable, user-friendly, and rapid testing devices to measure bilirubin and treat severe neonatal jaundice is critical in these regions. Research (57) indicates that integrating modern communication technologies, such as smartphones and Bluetooth, with devices enables developed sensors to achieve mobility, portability, and adaptability. This makes them effective even in remote, resource-scarce plateau regions.

## 3 Conclusions and future prospects

The literature reveals that no comprehensive diagnostic and treatment standards for neonatal hyperbilirubinemia exist in plateau regions. Current diagnostic criteria for neonatal hyperbilirubinemia are based on research data from lowland areas, and whether their intervention thresholds apply to high-altitude regions requires further investigation. Some scholars argue (22) that globally adopted diagnostic and treatment standards for neonatal hyperbilirubinemia inadequately account for the unique challenges of resource-limited settings. These include common high-altitude issues such as inadequate medical equipment (e.g., unstable phototherapy devices) and high prevalence of underlying conditions (e.g., infections, hemolysis). Consequently, diagnostic and treatment thresholds should be adjusted based on the characteristics of high-altitude newborns, taking into account regional environmental and resource considerations. Therefore, from the above discussion, we can observe that neonatal hyperbilirubinemia in high-altitude regions exhibits distinct clinical characteristics: early onset, rapid progression, high proportion of severe cases, and complex aetiology. Compounded by the adverse effects of the plateau environment, economic deprivation, lifestyle habits, and population genetic variations, this cohort requires not only locally adapted serum bilirubin detection methods (such as microfluidic chip CE/FA technology, skin photometry using vanadate oxidase, and neonatal-specific point-of-care testing devices), but also confronts numerous challenges in strategy implementation.

Regarding neonatal hyperbilirubinemia in high-altitude regions—a topic of significant research value in the medical field—future studies should focus on the following key directions: First, to address the complex effects of high-altitude geography and population characteristics on neonatal bilirubin metabolism, priority should be given to conducting large-scale, multi-centre studies in Tibet. Integrating medical resources to obtain representative data will enable systematic monitoring of bilirubin dynamics, establishing region-specific reference ranges and precise intervention thresholds to provide scientific guidance for clinical practice. Simultaneously, leveraging gene sequencing and bioinformatics technologies, we must delve into the genetic underpinnings and adaptive genetic alterations related to bilirubin metabolism in high-altitude populations. Identifying genetic biomarkers for disease risk will support early, precise prediction and personalised prevention and treatment. Additionally, portable rapid diagnostic devices optimised for high-altitude environments must be developed, balancing compact portability, rapid accuracy, and stability in harsh conditions to overcome diagnostic challenges caused by scarce medical resources and poor transportation. Finally, a long-term follow-up system should be established in collaboration with local health institutions. Standardised assessment tools will be used to regularly monitor the neurodevelopmental status of affected children, elucidating the long-term impact mechanisms of hyperbilirubinemia to inform optimised intervention strategies and improve outcomes.

In summary, neonatal hyperbilirubinemia (NHB) is a globally prevalent neonatal condition directly linked to fatal health outcomes. Survivors may also face irreversible long-term adverse consequences such as hearing impairment and intellectual disabilities. This article, grounded in the perspective of medical laboratory science, systematically reviews the specific challenges and appropriate technologies for laboratory diagnosis in this population. Its core objective is to provide targeted guidance for clinical laboratory professionals in high-altitude regions, thereby supporting the precise screening and standardised intervention for neonatal hyperbilirubinaemia in these areas. Ensuring all newborns with severe hyperbilirubinaemia or at high risk thereof receive timely, effective treatment and appropriate follow-up. This strategy will contribute to improving global child health development while precisely benefiting the health and wellbeing of residents in medically underserved areas such as remote high-altitude regions.
