# Supplementary material for: Advances in laboratory diagnosis of neonatal hyperbilirubinemia and peculiarities in plateau regions: a review of evidence
Source: Front Pediatr. 2026 Apr 9;14:1782889. doi: 10.3389/fped.2026.1782889 (PMC13102838; doi:10.3389/fped.2026.1782889)
Supplement: Supplementary file 1 [file Datasheet1.pdf]

Supplementary Material

In the Supplementary Materials, we have included translations of key excerpts from Chinese policy documents and a locally developed assessment scale to provide necessary context for the study's background and methodology. These are clearly cited and annotated to facilitate review.

1 Supplementary Note

Note 1 : Refrence 5: Pingcuo YJ, Wang DX, Qiong D, et al. Clinical analysis of 299 cases of Hyperbilirubineia in Tibetan neonates on plateau. *TIBETVSCIENCE AND TECHNOLOGY,* (2024) 46(02):56–60+7.[Article in Chinese].
Note 2 : Refrence 6: HM Y. A Study on the Incidence of Neonatal Hyperbilirubinemia and Hyperbilirubinemia Encephalopathy between the People´S Hospital of Naqu City and Shengjing Hospital. Shenyang: China Medical University(2019).[Article in Chinese]
Note 3 : Refrence 39:Li CG, Yao YL, Zhang GF. The risk prediction model of neonatal hyperbilirubinemia in Qinghai Plateau area was constructed based on UGT1A1 gene polymorphism.China Journal of Eugenies and Genetics (2025) 33(01):78–83. doi: 10.13404/j.cnki.cjbhh.2025.01.011.[Article in Chinese]

2 Supplementary Data

Note 1: Among the 299 cases of neonatal hyperbilirubinemia, 109 case (35.8%) were severe hyperbilirubinemia and 6 cases of acute bilirubinous encephalopathy , accounting for 5.7% of severe hyperbilirubinemia. The age of jaundice was 4 days (range: 1~26 days), the average age of admission was 7 days (range:1~30 days), and the pesk TSB was 342.5 ± 65.23 µmol/L(range 255.1–538.9).

Note 2: The NICU at Nagqu Municipal People's Hospital admitted 492 newborns in 2017, of whom 229 were diagnosed with hyperbilirubinaemia, representing an incidence rate of 46.54%. The NICU at Shengjing Hospital of China Medical University admitted 7,101 newborns in 2017, with 603 diagnosed with hyperbilirubinaemia, representing an incidence rate of 8.49%. This indicates that neonatal bilirubin levels in high-altitude regions are significantly higher than in lowland areas.

Note 3: Among 280 infants with neonatal hyperbilirubinaemia in the Qinghai plateau region studied, the c.211G>A variant was the most prevalent mutation site, occurring at a frequency of approximately 45.00% (126/280). This included 119 heterozygous variants (G/A or A/G) and 7 homozygous variants (A/A). Serum total bilirubin (TBil) levels were higher at the 211A/A locus and the 211G/A (or 211A/G) locus compared to the 211G/G (wild-type) locus. Other studies have also found that serum TBil levels in newborns with hyperbilirubinaemia carrying the c.211G>A mutation (including homozygotes and heterozygotes) in the Wuhan region were significantly higher than those in the wild-type. These observations suggest that variations in the UGT1A1 gene locus are present in neonatal hyperbilirubinaemia patients across different areas. Subsequent analyses may explore UGT1A1 gene polymorphisms among neonatal hyperbilirubinaemia patients of different races or ethnicities. This study further employed logistic multivariate regression analysis to confirm that the frequency of the c.211G>A variant and the A allele frequency constitute risk factors for neonatal hyperbilirubinaemia in the Qinghai Plateau region, indicating that the UGT1A1 gene c.211G>A mutation is a primary cause of neonatal hyperbilirubinaemia.
