# Supplementary material for: Advances in laboratory diagnosis of neonatal hyperbilirubinemia and peculiarities in plateau regions: a review of evidence
Source: Front Pediatr. 2026 Apr 9;14:1782889. doi: 10.3389/fped.2026.1782889 (PMC13102838; doi:10.3389/fped.2026.1782889)
Supplement: Supplementary file 2 [file Datasheet2.pdf]

高原藏族新生儿高胆红素血症299例临床分析

平措央吉 王东旭 琼达 泽碧 赵蓉
西藏自治区人民医院儿科，拉萨 850000

摘 要：**目的** 分析该院近2年新生儿高胆红素血症的发生情况、胆红素峰值及病因，为高原新生儿胆红素研究提供数据，为新生儿胆红素血症的预防及规范化管理提供依据。**方法** 回顾性总结了自2019年1月—2021年7月以黄疸相关主诉就诊于西藏自治区人民医院儿科的藏族近足月儿（>35周），描述了胆红素峰值分布情况、不同胎龄间的比较、伴发症分析及脑病患儿特征描述。**结果** 299例新生儿高胆红素血症病例数据中严重高胆红素血症109例（36.5%），急性胆红素脑病病例6例，占严重高胆红素血症的5.5%.发现黄疸日龄4天（范围：1~26d），平均入院日龄7天（范围:1~30d），TSB峰值为342.45±65.23 μmol/L。纳入病例中有91%伴有不同程度的感染，2例存在ABO自身免疫性溶血。**结论** 该研究数据展示了藏族近足月新生儿胆红素峰值分布情况，严重高胆红素血症患儿及脑病患儿的占比较高。因此，加大新生儿黄疸相关科普知识受众面，建立系统、全面的随访，为严重高胆红素血症的预防及规范化管理提供依据。

关键词：高原 新生儿高胆红素血症 脑病

Clinical Analysis of 299 Cases of Hyperbilirubinemia in Tibetan Neonates on Plateau

Pingcuo Yangji, Wang Dongxu, Qiong Da, Ze Bi, Zhao Rong

Department of Pediatrics, People's Hospital of Xizang Autonomous Region, Lhasa 850000, China

Abstract **Objective** To analyze the occurrence, peak bilirubin, and etiology of neonatal hyperbilirubinemia in our hospital in the past two years, to provide data for the study of high-altitude neonatal bilirubin, and to provide a basis for the prevention and standardized management of neonatal bilirubinemia. **Methods** The study retrospectively summarized the Tibetan near-term Tibetan infants (>35 weeks of gestation) who presented to the Department of Pediatrics of the People's Hospital of Xizang Autonomous Region from January 2019 to July 2021 with jaundice-related complaints, and described the distribution of bilirubin peaks, comparisons between different gestational ages, analysis of comorbidities, and characterization of children with encephalopathy. **Results** Among the 299 cases of neonatal hyperbilirubinemia, 109 cases (35.8%) were severe hyperbilirubinemia and 6 cases of acute bilirubinous encephalopathy, accounting for 5.7% of severe hyperbilirubinemia. The age of jaundice was 4 days (range: 1~26 days), the average age of admission was 7 days (range: 1~30 days), and the peak TSB was 342.45±65.23 μmol/L. Ninety-one percent of the included cases had varying degrees of infection, and two had ABO autoimmune hemolysis. **Conclusion** The data of this study showed the peak distribution of bilirubin in Tibetan near-term neonates, and the proportion of infants with severe hyperbilirubinemia and encephalopathy was relatively high, suggesting that the audience of neonatal jaundice related scientific knowledge should be expanded, and a systematic and comprehensive follow-up should be

收稿日期 2023-12-17 接受日期 2024-01-15
基金项目 西藏自治区人民医院院内项目（XZQYY2020022）
第一作者 平措央吉（1983-），女，西藏昌都人，本科，主治医师，主要研究方向为儿童支气管镜、儿童及新生儿重症、儿童呼吸道疾病等。E-mail:250689478@qq.com

(C)1994-2024 China Academic Journal Electronic Publishing House. All rights reserved. http://www.cnki.net

established to provide a basis for the prevention and standardized management of severe hyperbilirubinemia.

Keywords Plateau; Neonatal Hyperbilirubinemia; Encephalopathy

新生儿高胆红素血症是新生儿科常见疾病之一，新生儿出生后未规范监测和治疗时黄疸可能快速进展为严重高胆红素血症，并发急性胆红素脑病，甚至核黄疸，遗留中枢神经系统永久性损伤[1]。对新生儿胆红素水平进行动态监测、及时干预是防治脑病后遗症的重要手段。国内外已有大量资料提示胆红素水平有明显的种族、地域差异。从海拔因素考虑，西藏地区处于高海拔地区，高海拔地区胎儿在宫内长期处于相对缺氧的环境，引起了红细胞生成增多，新生儿出生过多的红细胞被破坏，胆红素生成增加，导致了新生儿体内胆红素水平比低海拔地区高[2]。从人种因素考虑，不同民族间决定胆红素代谢的基因存在差异，如基因差异导致的尿苷二磷酸葡萄糖醛酸基转移酶在胆红素代谢的作用下发生变化，另外机体缺氧程度及血红蛋白波动情况在不同种族间也存在差异[3]。不同地区、不同种族其新生儿黄疸血清胆红素峰值并不相同，故需要对我国各民族和各地区进行大样本新生儿黄疸流行病学调查，从而制定符合我国国情的新生儿黄疸诊断标准。已有研究提示高原地区新生儿高胆红素血症患病率较平原地区更高[4,5]，但相关数据没有更新，且西藏藏族新生儿高胆红素血症相关数据仍需要进一步积累。为此笔者回顾性总结了自2019年1月—2021年7月以黄疸相关主诉入院的近足月儿，描述了胆红素峰值分布情况、不同胎龄间的比较、伴发症分析及脑病患儿特征描述。

1 资料与方法

1.1 研究设计

本研究为单中心回顾性研究，回顾了自2019年1月年至2021年7月以黄疸相关主诉于西藏自治区人民医院儿科住院治疗的新生儿病例作为研究对象。

纳入标准：⑴ 胎龄≥35周；⑵ 父母均为藏族且长期居住于高海拔地区；⑶ 以发现皮肤黄染为主要主诉入院。

排除标准：⑴ 严重遗传代谢性疾病、胆道畸形问题；⑵ 无光疗前血液胆红素数据；⑶ 以直接胆红素增高为主的胆汁淤积症。

1.2 资料收集

母亲信息包括：民族，年龄，孕期所在地海拔高度，胎次、产次。

新生儿信息包括：出生时间、出生体重、入院体重、胎龄、性别、分娩方式、1分钟、5分钟Apgar评分、出现黄疸日龄、入院日龄、出院日龄、出院诊断。检验检查指标包括肝功能、血常规、感染指标、胸片、头颅MRI信息。回顾患儿现病史信息，若黄疸未接受光疗，则取住院后光疗前的第一次血清总胆红素（*Total serum bilirubin*, TSB）值，若在外院接受治疗，则取外院治疗前的TSB，并记录同时测得的血红蛋白相关指标及住院期间最高的CRP、PCT值辅助判断感染严重程度。

1.3 诊断标准及定义

根据我国《新生儿高胆红素血症诊断和治疗专家共识》[6]进行诊断及严重程度分类。

⑴ 新生儿高胆红素血症：以未结合胆红素升高为主，且TSB水平超过光疗参考曲线第95百分位。

⑵ 严重程度分级：重度高胆红素血症：342μmol/L（20 mg/dL）＜TSB峰值≤427μmol/L（25 mg/dL）；极重度高胆红素血症：427 μmol/L（25 mg/dL）＜TSB峰值≤510μmol/L（30 mg/dL）；危险性高胆红素血症：TSB峰值＞510 μmol/L（30 mg/dL）。本研究将以上3种高胆红素血症统称为严重高胆红素血症。

⑶ 急性胆红素脑病：急性胆红素脑病主要基于临床诊断，多见于胆红素指标高（TSB＞342μmol/L），和/或上升速度快（＞8.5μmol/L）的病人。

急性胆红素脑病患儿存在不同程度的中枢神经系统症状，包括早期肌张力减低、嗜睡、尖声哭、吸吮差，及后期出现肌张力增高、角弓反张、激惹、发热、惊厥、严重者可致死亡。但部分患儿可能缺乏典型症状，仅表现为呼吸暂停、循环呼吸功能急剧恶化等。

1.4 统计学分析

计量资料中符合正态分布的计量资料以均数±标准差（$\bar{x}\pm s$）表示，组间比较采用单因素方差分析。偏态分布计量资料以中位数（范围）表示，计数资料以例数和百分率(%)表示。用于比较严重高胆红素血症

(C)1994-2024 China Academic Journal Electronic Publishing House. All rights reserved. http://www.cnki.net

与一般高胆红素组基线数据的方法包括使用卡方检验来比较分类变量，对于连续变量，根据数据特点采用方差分析（One-way ANOVA）或Kruskal-Wallis检验。胆红素指标与血红蛋白的相关性用相关性研究。使用R（version 4.3.1）软件进行统计学分析，P<0.05为差异有统计学意义。

2 结果

2.1 总体情况

2019年1月—2021年7月西藏自治区人民医院儿科共收治因黄疸为主要主诉住院病例324例，根据纳排标准排除25例病人：父母非藏族病人4例，非新生儿期住院者4例，胆汁淤积症患儿1例，胎龄小于35周者16例，最终纳入299例新生儿高胆红素血症病人，其中严重高胆红素血症109例，占纳入病例的36.5%，急性胆红素脑病病例6例，占严重高胆红素血症的5.5%。

2.2 新生儿高胆红素血症患儿特征

299例新生儿高胆红素血症患儿均生活在海拔3500m以上地区，平均胎龄38.2±1.3周，其中胎龄介于35~37周的早产儿29例（9.7%），平均出生体重2932±492g，男性161例（53.8%）。剖宫产出生24例（30.4%）。发现黄疸日龄4天（范围：1~26d），平均入院日龄7天（范围:1~30d）。纳入的299例病例TSB峰值为342.45±65.23（范围:255.1~538.9）μmol/L（表1）。图1展示了纳入病例的TSB峰值分布情况。在胎龄35~<36周、36~<37周、37~<38周、≥38周间比较TSB峰值无显著差异，P值0.933。

表1 一般情况

Table 1 General Conditions of

n	所有患儿	TB ≤342μmol/L	TB>342μmol/L	P值
	299	192	107	
胎龄，周	38.23（1.29）	38.25（1.31）	38.19（1.26）	0.718
出生体重，g	2931.64（491.76）	2898.85（489.21）	2990.18（493.14）	0.124
入院体重，g	2905.56（515.31）	2873.71（495.82）	2962.43（546.13）	0.154
男性	161（53.8）	101（52.6）	60（56.1）	0.648
剖宫产出生	24（30.4）	16（32.7）	8（26.7）	0.757
1分钟Apgar≤7	4（1.3）	4（2.1）	0（0.0）	0.328
5分钟Apgar≤7	0（0.0）	0（0.0）	0（0.0）	-
母亲年龄，岁	30.27（5.00）	30.33（4.90）	30.17（5.23）	0.891
第一胎第一产	31（10.4）	22（11.5）	9（8.4）	0.685
所在地海拔，m	3854.11（345.55）	3831.15（325.47）	3895.33（377.02）	0.124
所在地海拔高于4000m	61（20.4）	33（17.2）	28（26.2）	0.09
发现黄疸日龄，天	3.64（3.18）	3.80（3.37）	3.36（2.83）	0.256
入院日龄，天	6.83（5.05）	6.92（5.46）	6.66（4.22）	0.679
胆红素脑病	6（2.0）	0（0.0）	6（5.7）	0.004
间接胆红素峰值，μmol/L	328.95（66.64）	282.18（27.31）	390.42（51.32）	<0.001
总胆红素峰值，μmol/L	342.45（65.23）	294.48（23.83）	404.32（46.89）	<0.001
血红蛋白，g/L	177.38（23.34）	175.71（23.77）	180.38（22.36）	0.099
红细胞压积，%	50.71（6.11）	50.30（5.90）	51.52（6.47）	0.115

注：连续分布数据以均数（标准差）表示，分类数据以例数（%）表示。
Note:Continuous distribution data are exrresced as mean±standard deviation, and categorical dataare expressed as number of caces (%).

(C)1994-2024 China Academic Journal Electronic Publishing House. All rights reserved. http://www.cnki.net

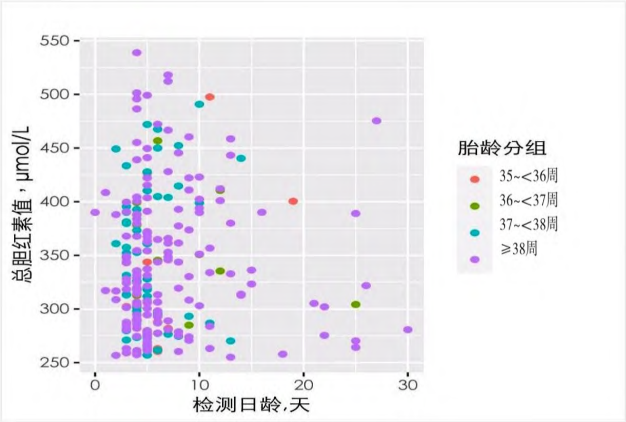

图1 不同胎龄不同检查日龄血清胆红素峰值分布图
Figure 1 The peak distribution of serum bilirubin at different gestational ages and different examination days

分析伴发症提示因皮肤黄染为主诉入院的新生儿中有272例(91%)合并了不同程度的感染，其中29例存在重症感染(21例为新生儿败血症、5例为新生儿坏死性小肠结肠炎、3例为颅内感染)。6例伴有头皮血肿、4例轻度窒息、3例摄入不足、2例合并ABO溶血。本研究同时收集了胆红素测定同期的血红蛋白、红细胞压积指标，提示血红蛋白均值177.38±23.34g/L，红细胞压积50.71±6.11%，单独比较血红蛋白与血清总胆红素相关性提示相关指数为0.0880。

入选病例中109例为严重胆红素血症，有19例完善了头颅MRI检查。6例诊断了急性胆红素脑病，脑病患儿总胆红素峰值均高于342μmol/L。比较严重胆红素血症组与新生儿胆红素血症组，提示2组间在新生儿胎龄、出生体重、黄疸出现日龄、血红蛋白等指标未见显著差异。

表2列出了6例胆红素脑病患儿信息，6例病人伴有不同程度的神经系统症状。编号1、2、4、6脑病患儿发现黄疸日龄为生后第4天，与首次就诊间隔2~7日不等。均伴发了新生儿肺炎。病例1、4头颅MRI结果正常，病例2、6拒绝完善头颅MRI。病例3在生后第一天发现黄疸，存在新生儿败血症，生后第3天于当地医院就诊后于生后11日龄时转诊至我院，其头颅MRI检查阴性。病例5发现黄疸日龄为22日龄，于家中观察5日后至我院就诊，头颅MRI检查阴性。6例病例均好转出院，出院时未见神经系统症状持续。

表2 胆红素脑病病人信息
Table 2 Information for patients with bilirubin encephalopathy

编号	胎龄，周	出生体重，g	性别	出生方式	出现黄疸日龄，天	入院日龄，天	异常神经系统症状	间接胆红素峰值，μmol/L	总胆红素峰值，μmol/L	头颅MRI
1	35	2000	男性	剖宫产	4	11	吃奶差、反应差、嗜睡、双眼凝视、哭声尖锐、四肢肌张力高	491.3	497.6	阴性
2	38	3100	男性	剖宫产	4	6	吃奶差、反应差、嗜睡、原始反射引出不全	438.4	472.2	拒绝检查
3	38	3200	男性	顺产	1	7	抽搐	490.6	512.2	阴性
4	39	3250	男性	顺产	4	7	吃奶差、双眼凝视、四肢肌张力低	448.1	466.7	阴性
5	39	2550	男性	顺产	22	27	吃奶差、抽搐、双眼凝视、角弓反张、原始反射引出弱	442.8	475.4	阴性
6	39	3000	男性	顺产	4	7	吃奶差、反应差、四肢肌张力低	494.0	518.1	拒绝检查

(C)1994-2024 China Academic Journal Electronic Publishing House. All rights reserved. http://www.cnki.net

3 讨论

本研究结果回顾性分析了299例新生儿高胆红素血症病例数据，其中严重高胆红素血症109例(36.5%)。急性胆红素脑病病例6例，占严重高胆红素血症的5.5%.发现黄疸日龄4天(范围：1~26d)，平均入院日龄7天(范围:1~30d)，TSB峰值为342.45±65.23 μmol/L。纳入病例中有91%伴有不同程度的感染，2例存在ABO自身免疫性溶血。

本研究结果提示，在3500m海拔以上生活的藏族近足月新生儿高胆红素血症患儿发现黄疸日龄为4天，与既往报道吻合[7-9]。值得注意的是自发现黄疸至门诊检查间间隔3天，提示对新生儿高胆红素血症在发现后仍存在延迟就诊的情况。一般情况下，我国顺产出生的足月儿生后3天，剖宫产出生的足月儿在生后5天时将随母亲出院，提示出院前密切监测患儿胆红素水平、出院后加强后续继续监测黄疸情况、及时识别严重高胆红素血症的高危因素、加强黄疸相关科普知识的宣教均非常重要。早期发现及早期治疗对新生儿高胆红素血症，预防胆红素中毒性脑病至关重要。

目前急性胆红素脑病诊断及临床分期是基于临床的诊断，但头颅MRI仍是诊断胆红素脑病的重要辅助检查[6]。本研究结果回顾性分析了299例新生儿高胆红素血症病例数据，其中严重高胆红素血症109例(36.5%)。其中仅有19例在住院期间完善了头颅MRI检查6例胆红素脑病病例中有4例在住院期间行头颅MRI检查。由此可见,严重高胆红素血症病例头颅MRI检查率较低。目前部分妇幼保健类医院尚没有开展MRI检查，住院期间头颅MRI检查对评估此类患儿预后非常重要，应创造条件完善头颅MRI检查，应加强产科及新生儿科医师对高胆红素血症的高危因素的识别与诊断能力，加强完善相关检验检查，为制订进一步的随访方案提供依据[6,10,11]。

本研究中提示胆红素脑病患儿6例，占严重高胆红素血症的5.5%.提示胆红素脑病患病率高于我国平原地区数据[7,12]，但由于本研究为单中心回顾性数据，需要进一步通过前瞻性多中心研究数据进一步明确。6例患儿在出院时无神经系统症状，但由于远期随访治疗缺乏，目前无法证实是否遗留神经系统后遗症。

胆红素脑病发生的危险因素包括小胎龄、围生期感染、溶血性疾病、出生时创伤(头皮血肿)、红细胞增多症等[13]。本研究提示血红蛋白指标与总胆红素相关性低，提示在高原地区，血红蛋白的增高并不是新生儿出现黄疸的主要影响因素。本研究纳入病例多伴发不同程度的感染，提示加强母亲围产期保健及新生儿早期管理重要性。尽管生后首日出现黄疸是严重高胆红素血症的危险因素之一[11]，但本研究中2例生后首日出现黄疸的ABO溶血患儿并未发展为严重高胆红素血症，可能与这两例患儿干预及时相关。这从另一个角度凸显了对新生儿黄疸的及时管理及随访的重要性。

本研究有一定局限性。首先，本研究为单中心回顾性研究，无法代表高原地区整体水平。第二，本研究纳入患儿为以黄疸为主诉入院的患儿，可以体现自发现黄疸至就诊时间的时间差异，且疾病相对较轻微，但未纳入危重患症且在住院期间伴发黄疸的人群。第三，我院新生儿听力筛查自2021年开始常规开展[14]，在本研究中无数据提供，且对于严重高胆红素血症的患儿，缺乏远期随访数据。但本研究查询了患儿在出院时的症状及查体，需要后期进一步开展随访工作明确高原地区新生儿胆红素脑病的患病情况。

80%以上的新生儿会在生后出现不同程度的黄疸[11]，且新生儿黄疸在新生儿所有死亡原因的全球排名中仍处于前列[15]，提示新生儿黄疸在世界范围内仍是一个严重的问题，而早期的识别及及时干预可防治胆红素脑病的发生[1,16]。本研究数据展示了胆红素峰值分布情况，严重高胆红素血症患儿及脑病患儿的占比较高，提示应对这部分病人建立系统、全面的随访，为严重高胆红素血症的预防及规范化管理提供依据。

(下转67页)

(C)1994-2024 China Academic Journal Electronic Publishing House. All rights reserved. http://www.cnki.net

用研究 [J]. 辽宁中医药大学学报, 2022, 24(08): 182-185.
[8] 王昊奋,漆桂林. 知识图谱:方法、实践与应用 [M]. 北京:电子工业出版社,2019:24-25.
[9] 吕子畔,黄仲羽,刘凤斌,等. 基于知识图谱的名医经验传承模式探究 [J]. 世界科学技术-中医药现代化,2020,22(12):4200-4204.
[10] 晓勇.《四部医典》:走向国际视野的藏医药经典论著 [N]. 西藏日报(汉),2023-06-14(006).
[11] 许丽,焦博,赵章瑞. 基于TF-IDF的加权朴素贝叶斯新闻文本分类算法 [J]. 网络安全技术与应用,2021(11):31-33.
[12] 高佳希,黄海燕. 基于TF-IDF和多头注意力Transformer模型的文本情感分析 [J/OL]. 华东理工大学学报(自然科学版):1-8 [2023-09-21].
[13] 陈建峡,黄煜俊,曹国金,等. 基于知识图谱的司法案件可视化研究与实现 [J]. 湖北工业大学学报,2019,34(05):72-77.
[14] 关鹏,王曰芬. 基于LDA主题模型和生命周期理论的科学文献主题挖掘 [J]. 情报学报,2015,34(3):286-299.

编校 洛松拉措

(上接60页)

参考文献

[1] Olusanya B O, Kaplan M, Hansen T W R. Neonatal hyperbilirubinaemia: a global perspective [J]. Lancet Child Adolesc Health,2018,2(8):610.
[2] 吴明昌,邵隽一. 新生儿黄疸 [J]. 中华全科医师杂志,2010(12):885-886.
[3] Basak N,Thangaraj K. High-altitude adaptation:Role of genetic and epigenetic factors [J]. Journal of biosciences,2021(46):107.
[4] Leibson C,Brown M,Thibodeau S,et al. Neonatal hyperbilirubinemia at high altitude [J]. American journal of diseases of children ,1989,143(8):983-987.
[5] Moore L G, Newberry M A, Freeby G M, et al. Increased incidence of neonatal hyperbilirubinemia at 3, 100 m in Colorado [J]. American journal of diseases of children,1984,138(2):157-161.
[6] 杜立中,马晓路. 新生儿高胆红素血症诊断和治疗专家共识 [J]. 中华儿科杂志,2014,52(10):745-748.
[7] 李倩倩,董小玥,乔瑜,等. 江苏省13家医院新生儿严重高胆红素血症现状调查 [J]. Chinese Journal of Contemporary Pediatrics,2020,22(7):690-695.
[8] 石碧珍,陈兰,韩树萍,等. 健康新生儿经皮小时胆红素百分位列线图预测高胆红素血症的价值 [J]. 中国当代儿科杂志,2016,18(3):201-205.
[9] 丁国芳,张苏平,姚丹,等. 我国部分地区正常新生儿黄疸的流行病学调查 [J]. 中华儿科杂志,2000,38(10):624.
[10] 王晓明,陈丽英,吴敏芳. 胆红素脑病的磁共振影像诊断 [J]. 中国小儿急救医学,2009(6):524-525+617.
[11] 佚名. 美国儿科学会新生儿高胆红素血症临床指南修订:胎龄35周及以上新生儿高胆红素血症的管理 [J]. 中华新生儿科杂志(中英文),2023,38(9):513-524.
[12] 中国新生儿胆红素脑病研究协作组中华医学会儿科学分会新生儿学组. 中国新生儿胆红素脑病的多中心流行病学调查研究 [J]. Chinese Journal of Pediatrics,2012,50(5):331-335.
[13] RIORDAN S M,SHAPIRO S M. Review of bilirubin neurotoxicity i: molecular biology and neuropathology of disease [J]. Pediatr Res,2020,87(2):327-331.
[14] 佚名. 新生儿听力筛查技术规范 [J]. 中国妇幼保健,2005,20(4):390-391.
[15] HAN ke, CHEN Shimin, SONG yang, et al. Burden of pancreatitis and associated risk factors in china, 1990 to 2019:a systematic analysis for the global burden of disease study 2019 [J]. 中华医学杂志英文版,2022,135(11):1340-1347.
[16] 涂阳阳,刘璐,原新慧,等. 重度新生儿高胆红素血症并发急性胆红素脑病的影响因素分析 [J]. 中国小儿急救医学,2021,28(6):516-520.

编校 洛松拉措

(C)1994-2024 China Academic Journal Electronic Publishing House. All rights reserved. http://www.cnki.net
