# Supplementary material for: Advances in laboratory diagnosis of neonatal hyperbilirubinemia and peculiarities in plateau regions: a review of evidence
Source: Front Pediatr. 2026 Apr 9;14:1782889. doi: 10.3389/fped.2026.1782889 (PMC13102838; doi:10.3389/fped.2026.1782889)
Supplement: Supplementary file 3 [file Datasheet3.pdf]

分类号：R725.7　　　　　　　　　　　　　　单位代码：10159

密　级：公开　　　　　　　　　　　　　　　学　　号：2016120885

中国医科大学

硕　士　学　位　论　文

（临床医学硕士专业学位）

中文题目：那曲市人民医院与盛京医院新生儿高胆红素血症及胆红素脑病发生率的双中心研究

英文题目：A study on the incidence of neonatal hyperbilirubinemia and hyperbilirubinemia encephalopathy between the people´s hospital of naqu city and shengjing hospital

论文作者：杨红美

指导教师：李玖军　教授

学科专业：儿科学

完成时间：2019年2月

(C)1994-2021 China Academic Journal Electronic Publishing House. All rights reserved.　http://www.cnki.net

(C)1994-2021 China Academic Journal Electronic Publishing House. All rights reserved. http://www.cnki.net

中国医科大学硕士学位论文

那曲市人民医院与盛京医院新生儿高胆红素血症及胆红素脑病发生率的双中心研究

A study on the incidence of neonatal hyperbilirubinemia and hyperbilirubinemia encephalopathy between the people´s hospital of naqu city and shengjing hospital

论文作者 杨红美　　指导教师 李玖军教授

申请学位 医学硕士　　培养单位 第二临床学院

一级学科 临床医学　　二级学科 儿科学

研究方向 小儿急救

论文课题起止时间 2017 年 1 月—2019 年 2 月

论文完成时间 2019 年 2 月

中国医科大学（辽宁）

2019 年 2 月

中国医科大学学位论文独创性声明

本人郑重声明：本论文是我研究生老师李玖军指导下进行的一项独立研究工作和研究成果，论文中除加以标注的内容外，不包含其他人或机构发表或撰写的研究成果，也不包含我用来为获得其他学位的成绩。对这项研究的其他个人和集体贡献均已在文中得到了明确的解释和表达。我很清楚，这一声明的法律后果是我的责任。

论文作者签名：[illegible]

日期：2019 年 5 月 31 日

中国医科大学学位论文版权使用授权书

作者充分理解学校对学位论文保存和使用的规定，同意学校保留学位论文的原件、复印件和电子版，并向国家有关部门或机构寄送，允许查阅和借阅。我授权中国医科大学将本论文的全部或部分内容汇编相关数据库进行检索，并通过复印、缩印或扫描等方式保存和编译论文。

保密（），在（）年后解密适用本授权书。（保密：请在括号内划“√”）

论文作者签名：[illegible]　　指导教师签名：李玖军

日期：2019年 5 月 31 日　　日期：2019年 5 月 31 日

(C)1994-2021 China Academic Journal Electronic Publishing House. All rights reserved. http://www.cnki.net

摘 要

目的：统计中国医科大学附属盛京医院 NICU 及那曲市人民医院 NICU 新生儿的血胆红素水平及胆红素脑病患儿的发病率，并进行分析比较，以期得到世居海拔 4500 米藏族新生儿与平原地区汉族新生儿血胆红素水平及胆红素脑病发病率的差异，为高海拔地区临床上如何干预新生儿黄疸及胆红素脑病提供理论参考。

方法：1.将中国医科大学附属盛京医院（平原地区）NICU 2017 年 1 月 1 日至 2017 年 12 月 31 日及那曲市人民医院（高原地区）NICU 2017 年 1 月 1 日至 2017 年 12 月 31 日入院患儿中高胆红素血症和发生胆红素脑病者纳入本研究，并收集其相关资料。

2. 在其中选取病例，回顾性分析 2017 年 1 月至 2017 年 12 月间，中国医科大学附属盛京医院 NICU 收治的 603 例高胆红素血症患儿及 2017 年 1 月至 2017 年 12 月那曲市人民医院 NICU 收治的 229 例高胆红素血症患儿，记录各组患儿入院基本情况（胎龄，性别，血红蛋白值、红细胞数、入院时日龄、最高胆红素值、是否换血及是否患胆红素脑病等），比较高原地区及平原地区儿新生之间各项临床资料的差异。

3.应用 SPSS22.0 统计软件分析和整理数据。计量资料符合正态分布的表示为均数±标准差表示，非正态分布资料表示为中位数±四分位数间距表示。组间均数比较采用非配对样本 t 检验，样本构成比或率的比较采用卡方检验。

结果：1.那曲市人民医院 NICU 2017 年共收治新生儿 492 例，其中有 229 例诊断为高胆红素血症，发生率为 46.54%，16 例出现胆红素脑病症状，发生率为 6.99%；中国医科大学附属盛京医院 NICU 2017 年共收治新生儿 7101 例，其中有 603 例诊断为高胆红素血症，发生率为 8.49%，45 例出现胆红素脑病症状，发生率为 7.46%。

2.经过病例筛选，高原地区、平原地区共 832 例高胆红素血症新生儿入选，高原地区共有 229 例，其中女性 99 例，男性 130 例，男女比

(C)1994-2021 China Academic Journal Electronic Publishing House. All rights reserved. http://www.cnki.net

例约为 1.31:1。平原地区共有 603 例，其中女性 255 例，男性 348 例，男女比例约为 1.36:1。

3.高原地区中胎龄最小者 25 周，最大者 39 周，胎龄≤27 周胎龄<28 周 19 例（8.3%），≤28 周胎龄<29 周 49 例（21.4%），≤29 周胎龄<30 周 10 例（4.4%），≤30 周胎龄<31 周 12 例（5.2%），≤31 周胎龄<32 周 29 例（12.7%），≤32 周胎龄<33 周 43 例（18.8%），≤33 周胎龄<34 周 12 例（5.2%），≤34 周胎龄<35 周 42 例（18.3%），≤35 周胎龄<36 周 7 例（3.1%），≤38 周胎龄<39 周例（1.3%），≥39 周 3 例（1.3%）。出生体重 1000～3100g（中位体重 1900g）。平原地区中胎龄最小者 28 周，最大者 42 周，胎龄≤28 周胎龄<29 周 2 例（0.3%），≤29 周胎龄<30 周 3 例（0.5%），≤30 周胎龄<31 周 13 例（2.2%），≤31 周胎龄<32 周 14 例（2.3%），≤32 周胎龄<33 周 10 例（1.7%），≤33 周胎龄<34 周 23 例（3.8%），≤34 周胎龄<35 周 37 例（6.1%），≤35 周胎龄<36 周 35 例（5.8%），≤36 周胎龄<37 周 47 例（7.8%），≤37 周胎龄<38 周 91 例（15.1%），≤38 周胎龄<39 周 81 例（13.4%），≤39 周胎龄<40 周 101 例（16.7%），≤40 周胎龄<41 周 93 例（15.4%），≤41 周胎龄<42 周 47 例（7.8%），≥42 周 6 例（1.0%）。出生体重 970～5050g（中位体重 3100g）。

结论：高原地区新生儿胆红素水平较平原地区明显增高，但胆红素脑病发生率却相对较低，可能与两方面因素相关，其一，在高海拔地区，生长在子宫内的胎儿长期处于相对乏氧的状态，刺激促红细胞生成素增多，从而导致胎儿的红细胞生成增多，胎儿离开母体后，很快建立自主呼吸，使其血氧浓度提高，过多的红细胞被破坏，胆红素生成量明显增加，导致了高海拔地域新生儿体内胆红素水平比低海拔地区高；其次也可能是不同民族间决定胆红素代谢的基因存在差异，目前关于此方面的相关研究较少，有待进一步研究。

关键词：新生儿高胆红素血症；胆红素脑病；高海拔地区；人种差异；

(C)1994-2021 China Academic Journal Electronic Publishing House. All rights reserved. http://www.cnki.net

Abstract

Objective: To calculate the blood bilirubin level and the incidence of high bilirubin encephalopathy in the NICU of Shengjing Hospital affiliated to China Medical University and the NICU of Naqu People's Hospital,and to analyze and compare them in order to obtain Tibetans with an altitude of 4,500 meters.The differences in blood bilirubin levels and the incidence of hyperbilirubin encephalopathy between neonates and newborns in the Han area provide a theoretical reference for how to intervene in neonatal jaundice and hyperbilirubin encephalopathy in high altitude areas.

Methods: 1. Naqu City People's Hospital NICU received a total of 492 cases of neonates in 2017, of which 229 cases were diagnosed as hyperbilirubinemia, the incidence rate was 46.54%, 16 cases of high bilirubin encephalopathy symptoms, the incidence rate was 6.99% In the 2017, a total of 7101 neonates were enrolled in the NIKU Hospital of China Medical University. Among them, 603 were diagnosed with hyperbilirubinemia, the incidence rate was 8.49%, and 45 patients developed symptoms of hyperbilirubin encephalopathy. 7.46%.

2.Among the cases selected,a retrospective analysis of 603 children with hyperbilirubinemia admitted to the NICU of Shengjing Hospital affiliated to China National Medical University from January 2017 to December 2017 and the cases from January 2017 to December 2017 229 children with hyperbilirubinemia admitted to the People's Hospital of the City of Naqu,recorded the basic conditions of admission to each group(gestational age,gender,hemoglobin value,number of red blood cells,age at admission,highest bilirubin value,whether to change blood And whether or not suffering from bilirubin encephalopathy,etc.,compare the clinical data of the newborns in the plateau and plain areas.

3.Analyze and organize data using SPSS 17.0 statistical software.The measurement data is in accordance with the normal distribution and is expressed by the mean±standard deviation,and the non-normal distribution data is expressed by the

(C)1994-2021 China Academic Journal Electronic Publishing House. All rights reserved. http://www.cnki.net

median±quartile spacing.The mean-to-group comparison was performed using the unpaired sample t test,and the sample composition ratio or rate was compared using the chi-square test.

Results:1.Naqu City People's Hospital NICU received a total of 492 neonates in 2017,229 cases diagnosed as hyperbilirubinemia,the incidence rate was 46.54%,and 16 cases diagnosed as bilirubin encephalopathy,the incidence rate 6.99%;Shengjing Hospital NICU received a total of 7101 newborns in 2017,603 cases diagnosed with hyperbilirubinemia,the incidence rate was 8.49%,and 45 cases diagnosed as bilirubin encephalopathy,the incidence rate was 7.46%.

2.After case screening,a total of 832 neonates with hyperbilirubinemia were enrolled in the plateau and plain areas.There were 229 cases in the plateau,including 99 females and130 males,with a male to female ratio of 1.31:1.There are 603 cases in the plains,including255 females and 348 males.The male to female ratio is about 1.36:1.

3.In the plateau, the minimum gestational age was 25 weeks, the largest was 39 weeks, and the gestational age was 27 weeks of gestational age <28 weeks in 19 cases (8.3%), 28 weeks gestational age <29 weeks 49 cases (21.4%), 29 weeks The gestational age was <30 weeks in 10 cases (4.4%), 30 weeks gestational age <31 weeks 12 cases (5.2%), 31 weeks gestational age <32 weeks 29 cases (12.7%), 32 weeks gestational age <33 weeks 43 cases (18.8%), 33 weeks gestational age < 34 weeks 12 cases (5.2%), 34 weeks gestational age < 35 weeks 42 cases (18.3%), 35 weeks gestational age < 36 weeks 7 cases (3.1%)), 38 weeks gestational age <39 weeks (1.3%), 39 weeks 3 cases (1.3%). The birth weight is 1000 to 3100 g (median weight 1900 g). In the plain area, the minimum gestational age was 28 weeks, the largest was 42 weeks, and the gestational age was 28 weeks gestational age <29 weeks 2 cases (0.3%), 29 weeks gestational age <30 weeks 3 cases (0.5%), 30 weeks The gestational age was <31 weeks in 13 cases (2.2%), 31 weeks gestational age <32 weeks 14 cases (2.3%), 32 weeks gestational age <33 weeks 10 cases (1.7%), 33 weeks gestational age <34 weeks Twenty-three patients (3.8%), 34 weeks gestational age <35 weeks 37 cases (6.1%), 35 weeks gestational age <36 weeks 35 cases (5.8%),

(C)1994-2021 China Academic Journal Electronic Publishing House. All rights reserved. http://www.cnki.net

36 weeks gestational age <37 weeks 47 cases (7.8%)), 37 weeks gestational age <38 weeks 91 cases (15.1%), 38 weeks gestational age <39 weeks 81 cases (13.4%), 39 weeks gestational age <40 weeks 101 cases (16.7%), 40 weeks The gestational age was <41 weeks in 93 cases (15.4%), 41 weeks gestational age <42 weeks 47 cases (7.8%),42 cases in 6 cases (1.0%). The birth weight is 970 to 5050 g (median weight 3100 g).

Conclusion: The level of neonatal bilirubin in the highland area is significantly higher than that in the plain area, but the incidence of high bilirubin encephalopathy is relatively low. It may be associated with high-altitude fetuses in the uterus for a long time in a relatively hypoxic environment, stimulating erythropoietin production. , resulting in increased erythropoiesis. After the newborn leaves the mother, spontaneous breathing is established quickly, the blood oxygen concentration is increased, too many red blood cells are destroyed, and the amount of bilirubin is significantly increased, resulting in high-altitude newborns. The level of bilirubin is higher than that of low altitude; it may also be the difference in genes that determine bilirubin metabolism among different ethnic groups. At present, there are few related studies in this area, which need further study.

Key words:neonatal hyperbilirubinemia;bilirubin encephalopathy;high altitude;ethnic differences;

(C)1994-2021 China Academic Journal Electronic Publishing House. All rights reserved. http://www.cnki.net

英文缩略词

英文缩写	英文全称	中文全称
NICU	Neonatal Intensive care unit	新生儿加强监护病房
ABE	Acute bilirubin encephalopathy	急性胆红素脑病
TSB	Total serum bilirubin	血清总胆红素
Bf	Free bilirubin	游离胆红素
UCB	Unbound bilirubin	未结合的胆红素
BAEP	Brainstem auditory evoked potential	脑干听觉诱发电位

(C)1994-2021 China Academic Journal Electronic Publishing House. All rights reserved. http://www.cnki.net

目 录

1 前言......1

2 资料与方法......1

2.1 入选病例......2

2.2 诊断标准......2

2.3 排除标准......2

2.4 方法......2

2.4.1 入院病人疾病发生率......2

2.4.2 临床资料......2

2.5 统计方法......2

3 结果......3

3.1 两组高胆红素血症患儿的胆红素水平比较......3

3.2 高原地区与平原地区高胆红素血症与胆红素脑病发生率的比较......3

3.3 两组患儿临床基本资料的比较......4

4 讨论......6

本研究创新性的自我评价......9

参考文献......10

综述......12

致谢......18

个人简历......19

(C)1994-2021 China Academic Journal Electronic Publishing House. All rights reserved. http://www.cnki.net

1 前言

新生儿高胆红素血症在临床上很常见，新生儿普遍在分娩后 3 天呈现黄疸，它们中的大多数会在 7-10 天后褪色，这是正常生理现象，黄疸可能会自然消退；如果一些新生儿的病情迅速恶化，则需要住院治疗，如不积极正确处理，可造成严重的并发症，即胆红素脑病，导致神经系统永久性损害，对社会和家庭构成极大危害。依据 2004 年美国儿科学会的新生儿高胆红素血症诊疗方案，胆红素脑病主要为胆红素对各类神经核的毒性所导致的中枢神经系统的临床表现。分娩后 7 天出现的胆红素神经毒性表现称为急性胆红素脑病[1]（Acute bilirubin encephal opathy，ABE）。因此，对新生儿黄疸进行适当、合理、安全、有效的干预，减少不必要的医疗护理和医疗资源浪费，防止高胆红素脑病的发生，是目前国际医学界多年来努力的目标。对于高原地区，受地理环境、民族及区域影响，新生儿高胆红素血症发生率明显增加。国内资料表示[2]，位于海拔 3100m 地区出生的婴儿发生黄疸的概率比在 1600m 地区出生的婴儿高出两倍，可见高海拔地区新生儿体内胆红素的水平较平原地区高。另有研究显示，藏族新生儿高胆红素血症的胆红素高峰较汉族新生儿高，藏族新生儿胆红素峰值(300.3±15.0)μmol/L，汉族新生儿胆红素峰值(296.5±17.8)μmol/L，两民族新生儿高胆红素血症发生率却没有明显差别。这些研究都显示了民族、海拔等因素与新生儿黄疸的特点具有相关性。我国目前的诊断标准沿用的是欧美国家的新生儿高胆红素血症的标准，但是这一诊断标准来自于平原地区，能否适用于高海拔地区，还有待研究。

近半个多世纪，随着人们对黄疸的认识和重视，对以高胆红素血症入院的患儿进行合理、适时的干预后，胆红素脑病的发生率明显下降，提示它是可以有效防治的疾病[3]。本研究对海拔 4500 米高原藏族新生儿与平原地区汉族新生儿高胆红素血症及急性胆红素脑病患儿的临床资料进行回顾性分析，从而得出胆红素水平和胆红素脑病发病率的差异，为高海拔地区临床上如何干预新生儿黄疸及高胆红素脑病提供理论参考。

2 资料与方法

2.1 入选病例

本课题为回顾性研究，收集入院时间为 2017 年 1 月 1 日-2017 年 12 月 31 日的那曲市人民医院 NICU 和中国医科大学附属盛京医院 NICU 的病例资料，进行病例筛选以确定研究对象，并收集基本实验室结果、临床诊断和治疗数据等。

2.2 诊断标准

符合《2014 年新生儿高胆红素血症诊断和治疗专家共识》以及《实用新生儿学》中新生儿高胆红素血症患儿、急性高胆红素脑病的诊断标准的高胆红素血症的患儿。

2.3 排除标准

(1)合并颅内感染：颅内感染可能导致儿童的拒食、嗜睡、易怒和抽搐。此时，不可能区分这些症状与胆红素引起的颅内感染或神经损伤，这影响了诊断的准确性。

(2)存在染色体异常、先天畸形：染色体异常、先天畸形等异常生长发育过程可能影响胆红素诱导神经毒性的过程，可能影响胆红素脑病的诊断结果和胆红素脑病的分析。

2.4 方法

2.4.1 入院病人疾病发生率

收集那曲市人民医院及中国医科大学附属盛京医 2017 年 1 月 1 日至 2017 年 12 月 31 日入院的新生儿资料，将住院新生儿总人数，以及住院期间诊断为新生儿高胆红素血症、胆红素脑病的人数进行统计，以计算住院新生儿中高胆红素血症组和胆红素脑病疾病的发生率。

2.4.2 临床资料

经过病例筛选，记录患儿一下基本信息

（1）患儿基本信息，包括胎龄、性别、入院时日龄、是否换血及是否患胆红素脑病等临床资料。

（2）临床检验资料，包括血红蛋白值、红细胞数、最高胆红素值。

(C)1994-2021 China Academic Journal Electronic Publishing House. All rights reserved. http://www.cnki.net

2.5 统计方法

应用 SPSS22.0 统计软件分析和整理数据。计量资料符合正态分布的用均数±标准差表示，非正态分布资料用中位数±四分位数间距表示。组间均数比较采用非配对样本 t 检验，样本构成比或率的比较采用卡方检验。

(C)1994-2021 China Academic Journal Electronic Publishing House. All rights reserved. http://www.cnki.net

3 结果

3.1 两组高胆红素血症患儿的胆红素水平比较

高原地区高胆红素血症患儿胆红素水平为225.7-675.9（umol/L），中位胆红素369.5（umol/L），平原地区高胆红素血症患儿胆红素水平为165-680.6（umol/L），中位胆红素278.8(umol/L)，两地区相比较，高原地区患儿的血清胆红素水平高于平原地区患儿的血清胆红素水平（表3.1）。

表 3.1 不同海拔 TSB 的差异

	病例数	TSB（中值）	U	p
高原组	229	369.5	8798	0.001
平原组	603	278.8		

两组 TSB 比较，*P*＜0.05，差异有统计学意义。

3.2 高原地区与平原地区高胆红素血症与胆红素脑病发生率的比较

那曲地区人民医院NICU 2017年共收治新生儿492例，其中有229例诊断为高胆红素血症，发生率为46.54%，16例出现胆红素脑病症状，发生率为6.99%（注：高原地区新生儿胆红素脑病的发病率为相应疾病发病人数在诊断为高胆红素血症新生儿中的比例）；中国医科大学附属盛京医院NICU 2017年共收治新生儿7101例，其中有603例诊断为高胆红素血症，发生率为8.49%，45例出现胆红素脑病症状，发生率为7.46%（平原地区新生儿胆红素脑病的发病率为相应疾病发病人数在诊断为高胆红素血症新生儿中的比例）。高原地区与平原地区新生儿中胆红素脑病的发生率有显著差异(*p*=O．022)(表3.2.3)。

表 3.2 胆红素脑病发病率

	高原	平原	p 值
发病	16（0.0699）	45（0.746）	0.022
未发病	213（0.9331）	558（0.254）	

P＜0.05，两地区胆红素脑病的发病率有统计学意义。

(C)1994-2021 China Academic Journal Electronic Publishing House. All rights reserved. http://www.cnki.net

3.3 两组患儿临床基本资料的比较

依照纳入标准进行筛选，高原地区共有 229 例纳入研究，平原地区共有 603 例纳入研究；对两地区患儿的胎龄，性别，血红蛋白值、红细胞数、入院时日龄、最高胆红素值、是否换血及是否患胆红素脑病等基本资料进行统计（表 3.3.1）。

高原地区高胆红素血症患儿中有男婴 130 例（56.8%），女婴 99 例（43.2%），胎龄 25 周～39 周，出生体重 1000 克～3100 克，血红蛋白值 92～248g/L，红细胞数 2.44～6.84*10^12/L，胆红素值 225.7～675.9umol/L。

平原地区高胆红素血症患儿中有男婴 348 例（57.7%），女婴 255 例（42.3%），胎龄 28 周～42 周，出生体重 970 克～5050 克，血红蛋白值 56～230g/L，红细胞数 1.2～34.9*10^12/L，胆红素值 165～680.6umol/L（表 3.3.2）。

表 3．3.1 两组患儿临床基本资料的比较

	高原地区	平原地区	统计量 U	P
胎龄中值	31	38	1184.5	0.001
血红蛋白中值	184	169	10585.5	0.001
红细胞数中值	4.79	4.8	17915	0.001
胆红素高峰时日龄中值	3	4	11784	0.001
胆红素中值	369.5	278.8	8798	0.001

P＜0.01,差异有明显统计学意义。

(C)1994-2021 China Academic Journal Electronic Publishing House. All rights reserved. http://www.cnki.net

(C)1994-2021 China Academic Journal Electronic Publishing House. All rights reserved. http://www.cnki.net

4 讨论

在 NICU 病房中，新生儿高胆红素血症的患儿占绝大部分，若有高危因素存在而延误治疗可导致最严重的并发症，胆红素脑病的发生，胆红素是通过血红素加氧酶的协同作用降解血红素的产物，血红素加氧酶将血红素转化为胆绿素，而胆绿素还原酶则将胆绿素还原为 UCB（未结合的胆红素）。UCB 在多种组织和细胞中发挥细胞毒性作用，会引起中枢神经系统永久性的损伤，胆红素脑病给家庭和社会带来了沉重的负担，因而防治胆红素脑病是新生儿高胆红素血症医治的主要目标[4]。我国目前沿用的是欧美国家新生儿高胆红素血症的诊断标准，即达到了相应日龄所对应的光疗曲线干预标准。有学者指出，早产儿是发生高胆红素血症及胆红素脑病的高危因素，一旦发生黄疸，会相对较重及持续时间较长，若按上述诊断标准可能会出现漏诊或延误治疗，可能早产儿在黄疸的诊断上还应考虑日龄及体重等因素。同样有资料显示，欧美国家的新生儿血清胆红素的峰值为 85.5～102.6μmol/L，一般出现在生后第 2 天～第 3 天，我国足月新生儿总胆红素的峰值为 171.0～205.2μmol/L，一般出现在分娩后第 4 天～第 5 天，四分之一的健康足月新生儿总胆红素可能达到 205.2μmol/L～220.6μmol/L，这一比例也高于欧美国家的新生儿[5]，这一研究表明胆红素水平可能因不同地区、不同种族而有差异。对于＞35 周重症高胆红素血症高危因素的认识，美国儿科学会在 2004 年时指出，不同种族、不同地区新生儿血清胆红素值出现的时间及高峰值并不相同，东亚种族后裔就是发生高胆红素血症的高危因素之一[6]；刘俐[7]、D,Apolito 等[8]指出，白种人是发生新生儿高胆红素的低危人群，东亚地区,亚洲和美洲印第安足月新生儿高胆红素血症的发生率较高；这与 Maisels[9]的研究相一致。唐述文[10]报道，维吾尔族新生儿高胆红素血症的发生率低于汉族，这可能与其生活的环境、海拔高度甚至遗传基因有关。本研究结果显示，高原地区胆红素血症新生儿的胆红素水平较平原地区高，与之前的研究相符合。

多数研究认为血清胆红素水平越高，造成胆红素脑损伤的可能性越大[11]。国外有研究报道，Bf 比 TSB 能更好地预测胆红素细胞毒性[12]，可能由于技术因素，临床上尚未普遍应用 Bf 进行测定，刘丽娟等[13]研究提示，在无法检测 Bf 的情况下，TSB 仍然有其预测胆红素毒性价值，因为 TSB 与 Bf 是呈正相关的。

(C)1994-2021 China Academic Journal Electronic Publishing House. All rights reserved. http://www.cnki.net

国外[14]通过胆红素普查提出的近足月新生儿血清总胆红素水平“危急”级别为25mg/dl(426umol/L)，当血清胆红素值达到这一水平时需要积极处理，而我们国家新生儿胆红素水平远远高于此值，仍然应用上述标准可能会延误治疗，因此，结合我国国情制定“危急”级别的总胆红素水平和寻找一个安全的总胆红素值值得临床探讨。吕峻峰等[15]研究指出，新生儿胆红素脑病产生不仅仅取决于胆红素水平，还与胎龄、出生体质量、胆红素结合状态及其某些病理情况下如缺氧、酸中毒、低蛋白、感染等血脑屏障功能开放多种因素相关。还有研究指出，在胎龄≥35 周的新生儿，分娩后早期喂养状况可影响其体重下降水平，摄入不足时，体重可能会下降更多，且主要体现为细胞外液的丧失[16]。由于脑血管内皮细胞及其细胞间的紧密衔接对维持血脑屏障性能十分关键，细胞外液的减少会不会对此结构产生影响，进而影响血脑屏障功能,导致胆红素脑病的产生，本课题中，平原地区中胎龄≥35 周的新生儿占 83%，高原地区中胎龄≥35 周的新生儿占 5.7%，这也可能是造成两地区胆红素脑病发病率差异的因素，但目前关于此方面的研究较少，仍是一个值得商榷的问题。

结合本文中的数据得知，世居海拔 4500 米藏族新生儿血胆红素水平明显高于平原地区汉族新生儿血胆红素水平，且差异有显著统计学意义，按目前国内诊断高胆红素血症的标准，高原地区藏族新生儿患高胆红素血症的比例高于平原地区汉族新生儿；且高原地区高胆红素血症患儿的血红蛋白值较平原地区患儿的血红蛋白值水平高，这可能是由于进化而导致其更加适应缺氧环境有关，新生儿离开母体后，很快建立自主呼吸，使其血氧浓度得到提高，导致过多的红细胞被破坏，胆红素生成量增加，导致了新生儿体内胆红素水平比低海拔地域高。高原地区胆红素脑病发病率较平原地区低，可能与海拔、民族、缺氧适应导致血脑屏障功能增强相关。

其他影响因素包括胎龄、出生体重、血红蛋白、黄疸高峰日龄等两组间的差异均有显著统计学意义。高原地区新生儿的血红蛋白值明显高于平原地区的新生儿血红蛋白水平，通过血红蛋白水平比较可知高原地区对缺氧环境适应性强，上述情况的发生可能与高原地区人对缺氧环境适应性强及种族差异相关性大。

综上所述，用平原地区或低海拔地区的胆红素水平标准诊断高原地区尤其是超高海拔地区的藏族新生儿是否患有高胆红素血症是不科学的，高原地区新生儿

(C)1994-2021 China Academic Journal Electronic Publishing House. All rights reserved. http://www.cnki.net

高胆红素血症的诊断应充分考虑海拔及民族要素，并制定相应的标准以指导临床诊治。

(C)1994-2021 China Academic Journal Electronic Publishing House. All rights reserved. http://www.cnki.net

本研究创新性的自我评价

既往平原地区与高原地区高胆红素血症诊断均采用统一标准，本文章通过对两地区胆红素水平及胆红素脑病发病率进行对比，提出高原地区高胆红素血症诊断应考虑地区及民族因素，为其诊断及治疗提供更加合理的理论指导。

(C)1994-2021 China Academic Journal Electronic Publishing House. All rights reserved. http://www.cnki.net

参考文献

[1]American Academy of Pediatrics Subcommittee on Hyperbilirubinemis Management of hyperbilirubinemia in the newborn infant 35 or more weeks of gestation。Pediatrics 2004，114(1)：297-316.

[2]马红.青藏汉族早期新生儿高胆红素血症发病情况分析[J].海医药杂志,2012,42(10).

[3]Maisels MJ.Neonatal jaundice[J].Pediatrics in Review,2006,27(12):443.

[4]Bhutani V K，Donn S M，Johnson L H．Risk management of severe neonatal hyperbilirubinemia to prevent kernicterus[J]．Clin Perinatol，2005，32(1)：125—139．

[5]刘义．新生儿黄疸的评估与干预［Ｊ］．中华儿科杂志，2001,39(6):321-322.

[6]中华医学会儿科学分会新生儿学组．新生儿黄疸诊疗原则的专家共识[J]．中华儿科杂志，2010,48(9):685-686.

[7]刘俐.我国新生儿黄疸诊治现状和面临的挑战[Ｊ].中华新生儿科杂志 2009(24):198-202.，

[8]D,Apolito M,Marrone A,ServedioV,et al.Seven novei mutations of the UGT1A1 gene in patients with unconjugated hyperbilirybinemia[J].Haematologica,2007,92:133-134.

[9]Maisels MJ.Neonatology[M].5th ed.Philadelphia:Lippincott Williams＆Wilkins Pub,1999:765-819.

[10]唐述文．维汉族新生儿高胆红素血症发病情况分析[J]．中国新生儿科杂志，2012，27(1):49-50.

[11]杜琨，张路，吴玉芹，新生儿重度胆红素血症 176 例病因分析［Ｊ］．中国中西医结合儿科学，2009,1(6):555-556.

[12]Ahlfors CE Predicting bilirubin neurotoxicity in jaundiced newborns[J].CurrOpin Pediatr,2010,22(2);129-133.

[13]刘丽娟，朴梅花，李松.血清游离胆红素的相关因素的研究[J].中国新生儿科杂志，2009,24（1）；11-14.

[14]WennbergRP,AhlforsCE,AravkinAY.Intervention guidelines for neonatal hyperbilirubinemia;an evidence based quagmire[J].Curr Pharm Des,2009,15(25);2939-2945.

[15]吕峻峰，杨冰岩，王维琼，等．严重高胆红素血症新生儿急性胆红素脑病危险因素分析[J]．中国新生儿科杂志，2014,29(4):242-246.

[16]Iman Iskander，MD，RashaGamaleldin，MD，Salma E1 Hou—chi，et a1．Serum bilirubin

(C)1994-2021 China Academic Journal Electronic Publishing House. All rights reserved. http://www.cnki.net

and bilirubin / albumin ratio as pre—dictors of bilirubin encephalopathy. EJ"1. Pediatrics，2014，134(5)：e1330 一 e1339.

(C)1994-2021 China Academic Journal Electronic Publishing House. All rights reserved. http://www.cnki.net

综 述

世居高原藏族新生儿与平原地区汉族新生儿血胆红素水平及其与高胆红素脑病相关关系的研究

新生儿黄疸及高胆红素血症是新生儿期最为多见的一类表现，大约有85%以上的足月儿和大部分早产儿在新生儿期会出现暂时性总胆红素增高现象[1]。重度高胆红素血症患儿倘若没有接受及时的医治，会进展成为核黄疸和急性胆红素脑病，是造成新生儿死亡的主要因素，可终身致残[2]。国内外数据资料已证实，新生儿胆红素水平有明显的种族、地域差别[3]。而西藏地处高海拔地区，地理环境、人种与区域差异以及藏族群众的生活习惯等因素导致黄疸的发生与平原地区存在较大差别[4]。不同地域、不同种族新生儿高胆红素血症的血清胆红素峰值并不一致，各个地区使用同一诊断标准来区别生理性黄疸和病理性黄疸，从而进行人为干预似乎并不合理[5]，因此分析高原地区新生儿胆红素水平及其特点显得至关重要，本文对世居高原藏族新生儿与平原地区汉族新生儿血胆红素水平及其与胆红素脑病相关关系进行综述以期给临床提供部分参考。

1、新生儿胆红素的代谢特点

一是因为新生儿红细胞寿命短、红细胞数量相对较多以及旁路胆红素来源增加导致胆红素生成过多；二是由于出生时肝细胞内受体蛋白Y含量较少导致肝细胞摄取胆红素能力不足；三是肝细胞对胆红素的代谢能力较差；四是胆红素的肝外代谢，即肝肠循环增加：胆红素经过肠道排泄时，在小肠基本不吸收，当到达回肠末端及结肠时，又由于肠内较高活性葡萄糖醛酸苷酶，水解结合胆红素转换为未结合胆红素，在肠道内被重吸收经门静脉而达肝脏，使胆红素肝肠循环负荷增加，加重了胆红素的重吸收。与此同时，当患儿出现饥饿、缺氧、脱水、酸中毒、头颅血肿及颅内出血时，黄疸更有可能发生并加重原有黄疸。血清亚单位可透过血脑屏障，引起神经细胞的黄染，如大脑基底核，引发核黄疸，可留下严重的后遗症，严重者危及生命[6-7]。

(C)1994-2021 China Academic Journal Electronic Publishing House. All rights reserved. http://www.cnki.net

2、新生儿高胆红素血症的诊断标准

新生儿黄疸一般分为两大类，即生理性黄疸和病理性黄疸，临床上也将病理性黄疸称为高胆红素血症。新生儿出生后的胆红素水平是一个动态过程，在诊断高胆红素血症时应考虑胎龄、年龄和高危要素。对于胎儿年龄≥35 周的新生儿，目前使用的是美国的 Bhutani[8]，应用程序推荐的每小时胆红素线性图或光疗参考曲线作为诊断或干预标准的参考。高胆红素血症是指胆红素水平超过 95 百分位时发生的高胆红素血症，应予以干预。根据不同的胆红素水平，大于 35 周的新生儿高胆红素血症还可以分为：重度高胆红素血症：血清总胆红素峰值超过 342umol/L(20mg/dl)；极重度高胆红素血症：TSB 峰值超过 427umol/L(25mg/dl)和 TSB 峰值超过 510umol/L(30 mg/dl)的危险性高胆红素血症。这一诊断标准考虑到了新生儿的实际情况，应和了胆红素水平存在日龄、小时龄、民族等差异性的观点，是符合临床实际的。但值得商榷的是，这一诊断标准的光疗干预指标是来自于平原地区，是否适用于高海拔地区，还有待研究。

有研究指出，高海拔地区的藏族新生儿黄疸出现的时间、高峰值及不同海拔高度都存在差异，对于血清总胆红素值的干预值，高海拔地区与平原地区有无差异也有待考证。因此，高海拔地区的临床诊断不可能完全参照平原地区的标准，这是因为在高海拔、缺氧环境下，机体会出现一系列代偿反应，比如红细胞的增多，造成在同一时间内衰老红细胞破坏裂解生成的胆红素会明显增加。另外不同的种族，遗传基因存在差异，在胆红素代谢过程中，尿苷二磷酸葡萄糖醛酸基转移酶对其产生的作用可能会发生改变，从而影响胆红素的代谢。

3、新生儿高胆红素脑病的相关概念

3.1 分类：胆红素脑病（Bilirubin Encephalopathy）分为急性胆红素脑病和慢性胆红素脑病，前者是指一种细胞毒性的急性期表现，通常发生在生后 1 周，后者又称核黄疸，是指胆红素毒性对神经系统所致的慢性、终身性的临床后遗症[9]。

3.2 临床表现：急性 BE 的主要临床表现为嗜睡、肌肉张力轻度下降、活动性下降和轻微高音哭泣；然后出现易怒、高音哭泣、拒绝进乳、呼吸困难和肌肉张力增加，最后，它转为了肌肉张力的降低[10]。慢性 BE 可表现为明显增加肌肉张力，智力发育和运动发育落后、手足运动、牙釉质发育不良、咀嚼和吞咽困难、听力异常。

(C)1994-2021 China Academic Journal Electronic Publishing House. All rights reserved. http://www.cnki.net

3.3临床诊断：参考《实用新生儿学》中急性胆红素脑病的诊断标准[11]：血清TSB≥342umol/L，或生后72小时内≥255umol/L，且具备下列条件之一：①继高胆红素血症之后出现异常神经系统表现，如尖叫、拒乳、意识状态改变、凝视、肌张力异常、惊厥等，能排除新生儿缺血缺氧性脑病、遗传代谢性疾病、颅内出血、内环境紊乱、颅内感染等；②脑干听觉诱发电位异常，不能用其他原因解释:胆红素可堆积于神经细胞，可出现脑干听觉诱发电位(BAEP)的异常改变，而脑干和第8对脑神经对胆红素毒性作用尤为敏感，高频短声刺激诱发的神经电位是儿童听觉传导通路损伤的敏感指标。而BAEP持续异常表明预后不良；③胆红素脑病急性期表现仍在研究中，一些研究表明，胆红素脑病急性期表现为T1wI高信号，T2WI高信号或常见侵袭部位稍高信号。苍白球区对称性Tl高信号为相对特征体现[12]。慢性阶段主要表现为好发部位的T2对称性高信号影，与组织学上胆红素脑病晚期胶质增生一致。有研究表明，如果仅发生急性期T1高信号，而相应部位在慢性期未出现T2高信号，则预后良好[13-14]。

4、民族与种族

新生儿胆红素水平在不同民族和种族间存在较大的差别，有文献报道，在东亚，足月新生儿分娩后每小时胆红素增高的风险较白种新生儿高，白人和黑人足月新生儿出生后每小时胆红素上升的速率明显低于亚洲和美洲印地安人，其高胆红素血症的发生率也明显低于亚洲和美洲印地安人[15]。Beal等[16]研究发现，黑种人发生新生儿黄疸风险最低，亚洲人（中国、韩国、日本人）和美国印第安人新生儿血清游离胆红素的最高水平明显高于是高加索人和黑种人，其胆红素脑病发病率明显增加，病情也更加严重。可见不同民族、种族及地区发生新生儿高胆红素血症差异的原因很可能包括基因的差异。这与Maisels[17]的观点相同。刘俐[18]指出，东亚地区的新生儿高胆红素的发生率较白种新生儿高；这与D’Apolito等[19]的报道相一致。唐述文[20]报道，维吾尔族新生儿高胆红素血症的发生率低于汉族，与Carceller等[21]、王文辉等[22]报道相同。维吾尔族新生儿高胆红素血症发生率较低可能与其生活的环境、海拔高度甚至遗传基因有关。这些都说明新生儿黄疸发生率存在民族差异。

5、世居高原地区的藏族新生儿与平原地区汉族新生儿血胆红素水平

(C)1994-2021 China Academic Journal Electronic Publishing House. All rights reserved. http://www.cnki.net

存在差异

有文献报道，高原地区新生儿血胆红素的水平较平原地区高，如在海拔3100m地区出生的新生儿比在海拔1600m地区出生的新生儿发生黄疸的概率高两倍。另有研究显示，藏族新生儿高胆红素峰值较汉族新生儿高，藏族新生儿胆红素峰值(300.3±15.0)umol/L，汉族新生儿胆红素峰值为(296.5±17.8)umol/L；但是两者新生儿高胆红素血症发生率无明显差别，马红[23]等研究显示青海红十字医院地处海拔2261m，在高海拔地区，孕母长期处于缺氧环境，可能会对胎儿造成不利影响，如宫内的缺氧窒息、胎儿生长发育缓慢、继发感染等，都可能造成新生儿高胆红素血症发生率的增高。这些研究都显示了新生儿生理性黄疸的特点与海拔、人种等因素具有相关性。

6、不同地区新生儿高胆红素脑病发病率

丹麦和加拿大报道的胆红素脑病发生率大约在 2.33/10 万-1.27/10 万[24-25]。由于我国人口众多，地区之间的医疗资源分布很不均衡，亚洲人本身就是高胆红素血症的高危人群，推断高胆红素脑病和核黄疸的发生率应该高于西方国家，但是由于缺乏大样本，在流行病学调查中，没有相应的疾病报告系统，具体的发病情况并不清楚。

7、结语

重度高胆红素血症如不及时治疗，将发生核黄疸和急性胆红素脑病，遗留下来的神经系统损伤可能会终身致残，因此新生儿高胆红素血症的干预至关重要。因为高原地区地理环境、人种与地域差异以及藏族群众的生活习惯等因素的影响，导致体内胆红素水平与平原地区存在较大差别，使用统一标准对新生儿高胆红素血症进行干预似乎不是很合理，为防止过度浪费医疗资源，更好地规范临床诊治，尚需进一步进行此方面的钻研，为高海拔地区临床上如何干预新生儿黄疸及高胆红素脑病提供理论参考。

(C)1994-2021 China Academic Journal Electronic Publishing House. All rights reserved. http://www.cnki.net

参考文献

[1]中华医学会儿科学分会新生儿学组．中国新生儿胆红素脑病的多中心流行病学调查研究[J]．中华儿科杂志，2012,50（5）：331-335.

[2]陈惠金，金汉珍，黄德珉，等.实用新生儿学[M].第3版．北京：人民卫生出版社，2013:297-305.

[3]金汉珍，黄德珉，官希吉.实用新生儿学．第二版．北京：人民卫生出版社，1997，216.

[4]邵肖梅,叶鸿瑁,丘小汕.实用新生儿学(第4版)[M].北京:人民卫生出版社,2011:271.

[5]Avery GB．Neonatolqgy．5th ed，Philadelphia：Lippin—cott W iUiams&W ilkins Pub.1999.765-819.

[6]扎西卓玛,格桑央金,小巴桑.高原地区新生儿高胆红素血症蓝光箱治疗的护理体会[J].西藏科技，2015，5：41-42.

[7]王晓青.新生儿胆红素代谢特点及黄疸分类[J].中国乡村医生杂志，1998，5:12-13.

[8]Bhutani VK，Johnson L，Sivieri EM．Predictive ability of a predischarge hour—specific sernm bilirubin for subsequent significant hyperbilirn．binemia in healthy term and Real Term newbornsl J I．Pediatrias，1999，103：6-14.

[9]Gentile G．Picardi A，Capnhianchi A．et a1．A prospective study comparing quantitative cytomesalovirus polymerase chain reaction in plasma and pp65 antigenemia assay in monitoring patients after allogeneic stem cell transplantation．BMC Infect Dis．2006.6：167.

[10-11]邵肖梅，叶鸿瑁，丘小汕．实用新生儿学［M］．4版．北京：人民卫生出版社，2011：298.

[12]Paul G，Maarten L，Renate SL．Changes inglobuspalliduswith(Pre)termkernicterus[J]．Pediatrics，2003，I 12(6 Pt I)：1256-1263．

[13]Abdulhakim C，All Y，Sefer K．Hyperintense globlus pallidus on T1一weighted MR imaging in acute kernicterus：is it common or rare?[J]．Eur Radiol，2005，15(6)：1263—1267．

[14]Harris MC，Bernbaum jc，Polin JR，et a1．Developmental fol-low-up of breastfed term and near-term infants with marked hy-perbilirubinemia[J]．Pediatrics，2001，107(5)：1075—1080．

[15]Voutetakis A，Maniati-Chrisddi M，Kanaka--．Gantenhein C,et a1．Prolonged jaundice and hypothyroidism as the Presenting symp。toms in a neonate with a novel propl gene mutation{[Q83X)[J],．Endoerinology，2004，150(3)：257-264.

(C)1994-2021 China Academic Journal Electronic Publishing House. All rights reserved. http://www.cnki.net

[16]Beal AC，. Chou SC，Palmer R H，et a1.，The changing face of race：risk factors for neonatal hyperbihrubinemia[Jil．Pediatrics，2006，1 17：1618-1625．

[17]Maisels MJ.Neonatology[M].5th ed.Philadelphia:Lippincott Williams&Wilkins Pub,1999:765-819.

[18]刘俐.我国新生儿黄疸诊治现状和面临的挑战[J].中华新生儿科杂志,2009(24):198 202.

[19]D' Apolito M,Marrone A,ServedioV,et al.Seven novel mutations of the UGT1A1 gene in patientswithunconjugated hyperbilirubinemia[J].Haematologica,2007,92:133-134.

[20]唐述文.维汉族新生儿高胆红素血症发病情况分析[J].中国新生儿杂志，2012,27（1）：49-50.

[21]Carceller BA，Cousineau J,Delvin EE．Point of care testing；transcutaneous bilirubinometry in neonates[J]．Clin Biochem，2009，42(14)：3-9.

[22]王文辉，王雪莉．阿克苏地区1986-2005年的少数民族新生儿疾病构成及死因分析．当代医学，2009，15：162—163．

[23]马红.高海拔地区新生儿黄疸的病因特点及评估干预[J]；高原医学杂志，2002，12（4）：48-49.

[24]Sgro M，Campkll D．Shah V．Incidence and causes of severe neonatal hyperbilirubinemia in Canada．CMAJ．2006．175：587-590.

[25]Maimburg RD，Beeh BH，Bjerre IV，et a1．Obstetric outcome in Danish children with a validated diagnosis of kemietems．Acta Obstet Gynecol Seand．2009．88：101 I．1016．

(C)1994-2021 China Academic Journal Electronic Publishing House. All rights reserved. http://www.cnki.net

致 谢

本论文是在我的导师李玖军教授的悉心指导下完成的，导师渊博的知识，严谨的治学态度，精益求精的工作作风，诲人不倦的高尚师德，严以律己、宽以待人的崇高风范，朴实无华、平易近人的人格魅力对我影响深远。不仅使我树立了远大的学术目标、掌握了基本的研究方法，还使我明白了许多待人接物与为人处事的道理。本论文从选题到完成，每一步都是在导师的指导下完成的，倾注了导师大量的心血。在此，谨向导师表示崇高的敬意的衷心的感谢！

三年的时光转眼即逝，中国医科大学这个大家庭，让我感受到了家一样的温暖。还记得三年前刚刚入学的时候，我们带着对未来的憧憬步入了临床工作的岗位，在各位学姐及带教老师的帮助下，我们逐渐熟悉和掌握了临床上一些常见病和多发病的诊治，并且掌握了腰穿、骨穿等临床常用的操作技能，为今后的工作奠定了坚实的基础，我很感谢这个大家庭，希望她可以更加壮大！

最后感谢抽出宝贵时间参加评审和答辩的各位老师！

杨红美

2019 年 2 月

(C)1994-2021 China Academic Journal Electronic Publishing House. All rights reserved. http://www.cnki.net

个人简历

姓名：杨红美

性别：女

民族：汉族

出生日期：1993 年 4 月 5 日

专业：小儿急救

学习经历：

2011 年 9 月-2016 年 6 月　沈阳医学院 临床医学专业

2016 年 9 月-2019 年 6 月　中国医科大学附属盛京医院 儿内科
