# Supplementary material for: Advances in laboratory diagnosis of neonatal hyperbilirubinemia and peculiarities in plateau regions: a review of evidence
Source: Front Pediatr. 2026 Apr 9;14:1782889. doi: 10.3389/fped.2026.1782889 (PMC13102838; doi:10.3389/fped.2026.1782889)
Supplement: Supplementary file 4 [file Datasheet4.pdf]

基于 *UGT1A1* 基因多态性构建青海高原地区新生儿高胆红素血症风险预测模型

李存桂*，姚彦莉，张国斐，侯培茹，王 斐，杨文静
（青海红十字医院儿科，青海西宁 810000）
*通讯作者：licungui@aliyun.com

【摘要】**目的** 基于尿苷二磷酸葡萄糖醛酸基转移酶 1A1（*UGT1A1*）基因多态性构建青海高原地区新生儿高胆红素血症风险预测模型。**方法** 将 2021 年 3 月至 2024 年 3 月青海红十字医院（高原地区）收治的 280 例高胆红素血症患儿作为观察组，同期 100 例无高胆红素血症的患儿作为对照组。两组患儿均采集静脉血并进行 *UGT1A1* 测序，分析 *UGT1A1* 基因各基因型频率、等位基因频率在两组患儿中的分布差异。基于青海高原地区新生儿高胆红素血症的危险因素构建列线图模型，并评估列线图模型预测效能。**结果** 观察组患儿 *UGT1A1* 基因变异位点中，以 c.211G>A 位点变异最为常见，约占 45.00%（126/280），且 c.211G>A 位点变异型频率及 A 等位基因频率均高于对照组（*P*<0.05）。进一步比较观察组 *UGT1A1* 基因 c.211G>A 位点不同基因型患儿血清总胆红素（TBil）水平，发现 211A/A 位点血清 TBil 水平最高，高于 211G/A（或 211A/G）位点、211G/G 位点（*P*<0.05）。对照组、观察组 TBil、间接胆红素（IDB）、天冬氨酸转氨酶（AST）、白蛋白（ALB）水平差异均具有统计学意义（*P*<0.05）。c.211G>A 位点变异型频率及 A 等位基因频率高，TBil、IDB、AST、ALB 水平高是影响青海高原地区新生儿高胆红素血症的影响因素。基于 c.211G>A 位点变异型频率及 A 等位基因频率等青海高原地区新生儿高胆红素血症的影响因素构建列线图模型，C-index 为 0.889（95%CI：0.807~0.938）。**结论** 青海高原地区新生儿高胆红素血症 *UGT1A1* 基因最常见的变异位点为 c.211G>A，且基于 c.211G>A 位点变异型频率及 A 等位基因频率等青海高原地区新生儿高胆红素血症的影响因素构建的列线图模型预测效能较好。

关键词：新生儿高胆红素血症；高原地区；基因多态性；预测模型
DOI:10.13404/j.cnki.cjbhh.2025.01.011

The risk prediction model of neonatal hyperbilirubinemia in Qinghai Plateau area was constructed based on *UGT1A1* gene polymorphism

LI Cungui, *YAO Yanli*, *ZHANG Guofei*, *HOU Peiru*, *WANG Fei*, *YANG Wenjing*
(*Pediatric Department of Qinghai Red Cross Hospital*, *Xining*, *Qinghai* 810000, *China*)

ABSTRACT **Objective** A risk prediction model of neonatal hyperbilirubinemia in Qinghai Plateau was constructed based on the polymorphism of uridine diphosphate glucuronidyltransferase 1A1 (*UGT1A1*) gene. **Methods** 280 children with hyperbilirubinemia admitted to the neonatal room of the Qinghai Red Cross Hospital (Plateau area) from March 2021 to March 2024 were selected as the observation group, and 100 children without hyperbilirubinemia were randomly selected as the control group during the same period. Venous blood was collected from both groups and uridine diphosphate glucuronidyltransferase 1A1 (*UGT1A1*) sequencing was performed to analyze the distribution differences of *UGT1A1* gene genotype frequency and allele frequency between the two groups. Based on the risk factors of neonatal hyperbilirubinemia in Qinghai Plateau area, a neomorph model was constructed and its prediction efficiency was evaluated. **Results** Among the *UGT1A1* gene variation sites in the observation group, the mutation of c.211G>A site was the most common, accounting for 45.00% (126/280), and the mutation frequency of c.211G>A site and the A allele frequency were higher than those in the control group (*P*<0.05). The serum total bilirubin (TBil) levels of *UGT1A1* gene c.211G>A locus in different genotypes in the observation group were further compared, and it was found that the serum TBil level at 211A/A locus was the highest, higher than that at 211G/A (or 211A/G) locus and 211G/G locus (*P*<0.05). There were significant differences in the levels of TBil, indirect bilirubin (IDB), aspartate aminotransferase (AST) and albumin (ALB) between control group and observation group (*P*<0.05). The frequency of c.211G>A locus variant and the frequency of A allele were high, and the levels of TBil, IDB, AST and ALB were the influential factors affecting neonatal hyperbilirubinemia in Qinghai Plateau area. A histogram model was constructed based on the influence factors of neonatal hyperbilirubinemia in Qinghai Plateau area, such as the variation frequency of c.211G>A locus and the frequency of A allele, the C-index was 0.889 (95%CI: 0.807-0.938), and the measured values were basically consistent with the predicted values. **Conclusion** The most common variation locus of *UGT1A1* gene in neonatal hyperbilirubinemia in Qinghai plateau area was c.211G>A, and the nomogram model built based on the variation frequency of c.211G>A locus and the frequency of allele of A was effective in predicting neonatal hyperbilirubinemia in Chinese plateau region.

KEY WORDS neonatal hyperbilirubinemia; plateau areas; gene polymorphism; prediction model

中国知网 https://www.cnki.net

高胆红素血症是新生儿期常见的疾病之一，血清血胆红素超过 17.1 μmol/L 即可确诊，常有皮肤黏膜、巩膜黄染及尿色、便色改变等表现，严重者可出现胆红素脑病、遗留神经系统后遗症[1-2]。最新研究表明，溶血性疾病、感染均可诱发新生儿高胆红素血症，但部分患儿完善相关检查后仍不能明确病因[3-4]。近年来研究[5]发现，不明原因的新生儿高胆红素血症往往与胆红素代谢障碍有关，且存在遗传学基础。既往因基因检测方法的限制，临床病因诊断相对困难，现已证实，与胆红素代谢相关的基因有尿苷二磷酸葡萄糖醛酸基转移酶 1A1（uridine diphosphate glucuronidyltransferase 1A1，*UGT1A1*）、血红素加氧酶 1 基因等，其中 *UGT1A1* 突变基因型最多，已达 100 余种[6-7]。*UGT1A1* 定位于 2q37，是人类体内唯一能催化未结合胆红素代谢的酶，其活性下降或缺失时直接影响胆红素合成，诱发胆红素代谢性疾病。*UGT1A1* 基因突变存在地域、种族或民族差异，本研究基于 *UGT1A1* 多态性，构建青海高原地区新生儿高胆红素血症风险预测模型，现报道如下。

1 资料与方法

1.1 一般资料

将 2021 年 3 月至 2024 年 3 月青海红十字医院（高原地区）新生儿室收治的 280 例高胆红素血症患儿作为观察组：男 158 例、女 122 例，胎龄 37~42 周、平均胎龄（38.30±1.50）周，同期 100 例无高胆红素血症的患儿作为对照组：男 52 例、女 48 例，胎龄 37~42 周、平均胎龄（38.50±1.20）周，两组患儿性别比、胎龄差异均无统计学意义（P>0.05）。纳入标准：胎龄 37~42 周，日龄 <28 d；出生体质量位于 2500~3999 g 区间；观察组患儿确诊为高胆红素血症[8]；患儿直系亲属签署研究知情同意书。排除标准：对照组患儿为出血性疾病、葡萄糖-6-磷酸脱氢酶（glucose-6-phosphate dehydrogenase，G6PD）缺乏等可能影响胆红素水平的疾病；合并先天性畸形、遗传代谢性疾病或染色体异常；使用过水杨酸类、磺胺类等影响胆红素代谢的药物；临床资料不齐全者。本研究经青海红十字医院伦理委员会审核通过（批号：2021027）。

1.2 方法

1.2.1 临床资料收集　记录患儿性别、民族、胎龄、出生体质量、分娩方式、喂养方式，入院日龄、血常规结果、肝功能指标及凝血功能指标。

1.2.2 *UGT1A1* 基因测序　采集患儿外周静脉血，严格按照全血 DNA 提取试剂盒使用说明提取基因组 DNA，TSND 2000 分光光度计（上海聚慕医疗器械有限公司）测定基因组 DNA 浓度，−20 ℃冰箱保存备用。设计 *UGT1A1* 基因聚合酶链反应（polymerase chain reactio，PCR）引物，采用 *Tap* DNA 聚合酶试剂盒对 DNA 样本 *UGT1A1* 基因外显子及其侧翼序列(上海华大基因科技有限公司）进行扩增，扩增后运用电泳仪（北京佳航博创科技有限公司）将 PCR 反应产物进行 DNA 朝正极的方向迁移，而后应用 ABI 3500 测序仪（美国 ABI 公司）Sanger 测序，试验所用试剂盒均购于上海酶联生物科技有限公司。

1.3 观察指标

（1）比较对照组、观察组 *UGT1A1* 基因分布情况；（2）比较对照组、观察组临床资料；（3）筛选青海高原地区新生儿高胆红素血症发生的影响因素；（4）构建青海高原地区新生儿高胆红素血症风险列线图模型并验证其预测效能。

1.4 统计学方法

采用 SPSS 26.0 统计学软件对患儿临床资料进行单因素分析，其中计量资料均以均数 ± 标准差（$\bar{x}\pm s$）描述，组间比较采用独立样本 t 检验，计数资料均以例（百分比）[n（%）]描述，组间比较采用 χ^2 检验或 Fisher 确切概率法；Logistic 多因素回归分析法筛选影响青海高原地区新生儿高胆红素血症发生的影响因素；R 3.5.3 软件构建青海高原地区新生儿高胆红素血症风险列线图模型，rms 程序包、Bootstrap 内部验证法评估列线图模型预测效能。以 P<0.05 提示差异有统计学意义。

2 结果

2.1 对照组、观察组 *UGT1A1* 基因分布情况比较

观察组患儿共检测到 c.211G>A、c.1091C>T、c.1456T>G 3 个 *UGT1A1* 基因变异位点，其中以 c.211G>A 位点变异最为常见，约占 45.00%（126/280）；对照组患儿共检测到 c.211G>A、c.1091C>T 2 个 *UGT1A1* 基因变异位点，其中 c.211G>A 位点变异[18.00%（18/100）]高于 c.1091C>T 位点变异[6.00%(6/100)]。观察组 c.211G>A 位点变异型频率及 A 等位基因频率均高于对照组（P<0.05），两组 c.1091C>T、c.1456T>G 位点分布差异无统计学意义（P>0.05），见表 1。进一步比较观察组 *UGT1A1* 基因 c.211G>A 位点不同基因型患儿血清总胆红素（total bilirubin，TBil）水平，发现 211A/A 位点血清 TBil 水平最高，高于 211G/A（或 211A/G）位点、211G/G 位点（P<0.05），见表 2。

表 1　对照组、观察组 *UGT1A1* 基因分布情况比较[n（%）]

UGT1A1 基因分布情况	对照组（n=100）	观察组（n=280）	χ^2	P
c.211G>A				
基因型频率			23.291	0.000
野生型（G/G）	82（82.00）	154（55.00）		
杂合变异型（G/A）	16（16.00）	119（42.50）		

中国知网 https://www.cnki.net

续表

UGT1A1 基因分布情况	对照组（*n*=100）	观察组（*n*=280）	χ^2	*P*
纯合变异型（A/A）	2（2.00）	7（2.50）		
等位基因频率			31.662	0.000
G	180（90.00）	392（70.00）		
A	20（10.00）	168（30.00）		
c.1091C>T				
基因型频率			0.251	0.616
野生型（C/C）	94（94.00）	259（92.50）		
杂合变异型（C/T）	6（6.00）	21（7.50）		
纯合变异型（T/T）	0（0.00）	0（0.00）		
等位基因频率			3.288	0.070
C	196（98.00）	532（95.00）		
T	4（2.00）	28（5.00）		
c.1456T>G				
基因型频率			—	0.111[a]
野生型（T/T）	100（100.00）	273（97.50）		
杂合变异型（T/G）	0（0.00）	7（2.50）		
纯合变异型（G/G）	0（0.00）	0（0.00）		
等位基因频率			—	0.112[a]
T	200（100.00）	553（98.75）		
G	0（0.00）	7（1.25）		

注：[a]为 Fisher 确切概率法计算。

表 2　观察组 *UGT1A1* 基因 c.211G>A 位点不同基因型患儿血清 TBil 水平比较（$\bar{x}\pm s$，μmol/L）

c.211G>A 位点基因型	例数	TBil
221G/G	154	220.95±8.52
221G/A（A/G）	119	230.25±7.55[a]
211A/A	7	242.55±5.62[ab]
F		60.766
P		0.000

注：[a]与 221G/G 比较，*P*<0.05；[b]与 221G/A（A/G）比较，*P*<0.05。

2.2　对照组、观察组临床资料比较

对照组、观察组 TBil、间接胆红素（indirect bilirubin，IDB）、天冬氨酸转氨酶（aspartate aminotransferase，AST）、白蛋白（albumin，ALB）水平差异均具有统计学意义（*P*<0.05），血红蛋白（haemoglobin，Hb）、白细胞计数（white blood cell count，WBC）、网织红细胞计数（reticulocyte cunt，Rtc）等临床资料比较差异无统计学意义（*P*>0.05）。见表 3。

2.3　青海高原地区新生儿高胆红素血症的影响因素

c.211G>A 位点变异型频率及 A 等位基因频率高，

表 3　对照组、观察组临床资料比较

临床资料	对照组（*n*=100）	观察组（*n*=280）	t/χ^2	*P*
男[*n*（%）]	52（52.00）	150（53.57）	0.073	0.787
民族[*n*（%）]				
藏族	50（50.00）	135（48.21）	0.094	0.759
回族	25（25.00）	82（29.29）	0.669	0.413
土族	15（15.00）	43（15.36）	0.007	0.932
其他	10（10.00）	20（7.14）	0.827	0.363
胎龄（周）	38.50±1.20	38.30±1.50	1.203	0.230
出生体质量（g）	3015.50±12.50	3016.02±10.62	0.401	0.689
分娩方式[*n*（%）]			0.195	0.659
阴道分娩	60（60.00）	175（62.50）		
剖宫产	40（40.00）	105（37.50）		

中国知网　https://www.cnki.net

续表

临床资料	对照组（n=100）	观察组（n=280）	t/χ^2	P
喂养方式[n（%）]			0.722	0.697
母乳	40（40.00）	122（43.57）		
代乳品	24（24.00）	70（25.00）		
混合喂养	36（36.00）	88（31.43）		
入院日龄（d）	6.20±1.50	6.40±1.20	1.336	0.182
入院血常规（$\bar{x}\pm s$）				
Hb（g/L）	112.85±12.25	113.62±12.50	0.532	0.595
WBC（$\times10^9$/L）	10.62±2.45	10.75±2.50	0.449	0.654
Rtc（$\times10^9$/L）	0.15±0.02	0.16±0.05	1.944	0.053
TG（mmol/L）	0.89±0.12	0.92±0.19	1.477	0.141
TC（mmol/L）	3.22±0.62	3.35±0.75	1.554	0.121
入院肝功能指标（$\bar{x}\pm s$）				
TBil（μmol/L）	100.00±8.45	220.44±12.50	89.302	0.000
IDB（μmol/L）	50.00±3.26	180.62±15.17	85.335	0.000
AST（U/L）	30.58±6.25	36.65±4.15	10.878	0.000
ALT（U/L）	10.50±2.05	10.75±1.98	1.074	0.284
入院凝血功能指标（$\bar{x}\pm s$）				
APTT（s）	40.50±4.50	40.00±3.95	1.047	0.296
PT（s）	13.40±2.50	13.20±2.00	0.801	0.423
FIB（g/L）	2.05±0.45	2.08±0.51	0.520	0.603
ALB（g/L）	35.25±4.25	40.50±3.02	13.311	0.000

注：Hb、WBC、Rtc、TG、TC 分别为血红蛋白、白细胞计数、网织红细胞计数、甘油三酯、总胆固醇，TBil、IDB、AST、ALT 分别为总胆红素、间接胆红素、天冬氨酸转氨酶、丙氨酸转氨酶，APTT、PT、FIB、ALB 分别为活化部分凝血活酶时间、凝血酶原时间、纤维蛋白原、白蛋白。

TBil、IDB、AST、ALB 水平高是青海高原地区新生儿高胆红素血症的影响因素。见表 4。

表 4　青海高原地区新生儿高胆红素血症的影响因素

自变量	β	SE	Waldχ^2	P	OR	95%CI
常数项	−53.026	0.811	20.252	0.000	—	—
c.211G>A 位点变异型频率	0.814	0.457	6.909	0.008	1.825	1.507~2.719
c.211G>A 位点 A 等位基因频率	0.821	0.451	6.792	0.000	1.907	1.726~2.784
TBil	0.738	0.438	6.628	0.001	1.756	1.536~2.505
IDB	0.716	0.418	6.680	0.000	1.749	1.585~2.445
AST	0.728	0.449	6.535	0.000	1.726	1.535~2.487
ALB	0.729	0.455	6.527	0.000	1.718	1.409~2.576

2.4　青海高原地区新生儿高胆红素血症风险列线图模型构建及验证

基于 c.211G＞A 位点变异型频率及 A 等位基因频率等青海高原地区新生儿高胆红素血症的影响因素构建列线图模型，见图 1；Hosmer-Lemeshow 检验结果显示，列线图模型实测值与预测值差异无统计学意义（χ^2=2.154，P>0.05），见图 2；受试者操作特征（receiver operator characteristic，ROC）曲线结果显示，列线图模型一致性指数（consistency index，C-index）为 0.889（95%CI：0.807~0.938），预测效能良好，见图 3。

3　讨论

高胆红素血症是新生儿黄疸最常见的表现形式，游离的胆红素透过血脑屏障进入脑细胞后，抑制其对氧的利用能力，导致脑细胞受损，严重者可致神经系统永久性损害，甚至死亡[9-10]。UGT 是胆红素代谢的关键酶，其中 *UGT1A1* 是由第 1 个外显子和 4 个共同外显子（2~5）组成的复合体，也是人类体内唯一能降低血清胆红素水平的酶，近年来研究发现，*UGT1A1* 不同位点变异使

中国知网 https://www.cnki.net

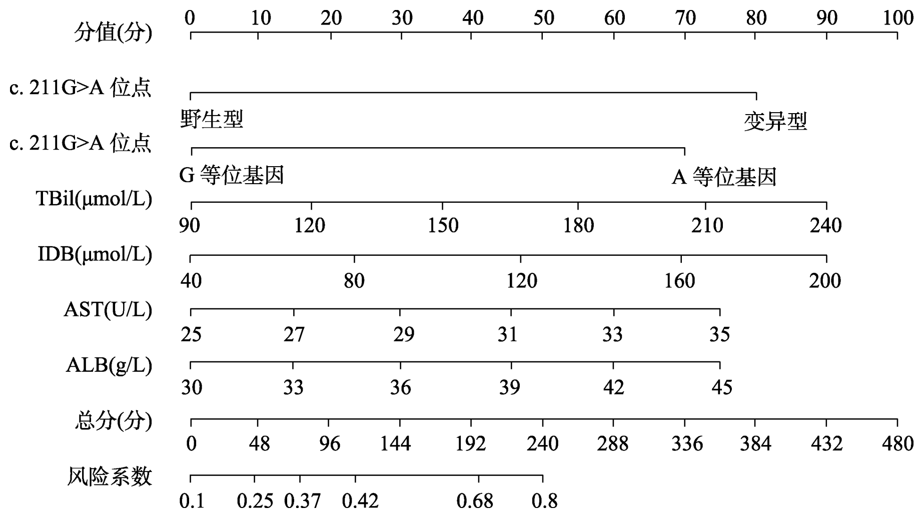

图 1　青海高原地区新生儿高胆红素血症风险列线图模型

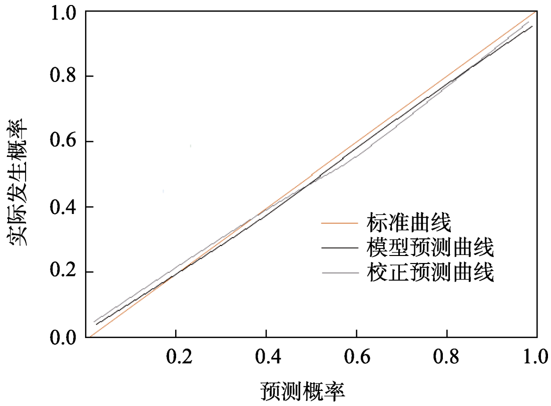

图 2　列线图模型内部验证的校准度（Hosmer-Lemeshow 检验）

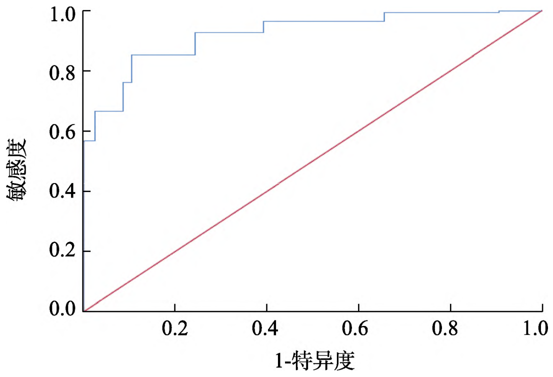

图 3　列线图模型预测效能（ROC 曲线）

UGT1A1 相关酶活性下降程度不同[11-12]。本研究对 280 例青海高原地区新生儿高胆红素血症患儿进行 *UGT1A1* 基因测序，共检出 c.211G>A、c.1091C>T、c.1456T>G 3 个 *UGT1A1* 基因变异位点，且均为外显子区域变异。此外，观察组 c.211G>A 位点变异型频率及 A 等位基因频率均高于对照组，提示 *UGT1A1* 基因变异位点 c.211G>A 可能是诱发青海高原地区新生儿高胆红素血症的重要因素。

c.211G>A 位于 *UGT1A1* 基因第 1 个外显子上，有研究[13]发现，c.211G＞A 变异后的表达产物可使蛋白质构象发生改变，降低 *UGT1A1* 活性，且纯合变异型时 *UGT1A1* 酶活性（22%）明显低于杂合变异型（71%）。本研究 280 例青海高原地区新生儿高胆红素血症患儿中，c.211G>A 为最常见的变异位点，其变异频率约为 45.00%（126/280），包含 119 例杂合变异（G/A 或 A/G），7 例纯合变异（A/A），且 211A/A 位点、211G/A（或 211A/G）位点血清 TBil 水平均高于 211G/G（野生型）；也有研究[14]发现武汉地区 c.211G>A 突变（包括纯合子、杂合子）新生儿高胆红素血症患儿血清 TBil 水平显著高于野生型。上述观点提示，不同地区间新生儿高胆红素血症患儿均存在 *UGT1A1* 基因位点变异，后续可分析不同种族或民族间新生儿高胆红素血症患儿间 *UGT1A1* 基因多态性。本研究进一步经 Logistic 多因素回归分析法证实，c.211G>A 位点变异型频率及 A 等位基因频率是影响青海高原地区新生儿高胆红素血症的危险因素，提示 *UGT1A1* 基因 c.211G>A 是诱发新生儿高胆红素血症的主要原因。

胆红素具有抗自由基、脂质过氧化的作用，TBil、IDB 过高时发挥细胞毒性作用，破坏细胞膜结构，干扰细胞能量代谢，继而损伤组织、器官[15-16]。肝脏担负着人体代谢、解毒及合成等重要生理功能，AST 是反映肝功能整体情况的重要指标，本研究结果“AST 是影响青海高原地区新生儿高胆红素血症”的原因可能在于肝脏是胆红素的主要代谢场所，TBil 过高会增加肝脏负担，同时对肝细胞产生一定的毒性作用，降低肝功能，故 AST 异常表达[17]。ALB 在维持机体营养状况、血浆渗透压、免疫功能等方面具有重要价值，既往研究[18]发现，ALB 升高后导致的肝功能异常是诱发新生儿高胆红素血症的独立危险因素，由此可见关注新生儿 ALB 动态变化有望降低高胆红素血症发生风险。也有研究[19-20]认为，多数患儿存在进行性肝细胞损伤及肝功能下降的情况，进食、消化能力相对更差，不利于肝脏合成 ALB，降低患儿凝血功能，后续可进一步分析高胆红素血症患儿肝功能与

中国知网　https://www.cnki.net

凝血功能的相关性。上述单一的指标作为诊断或预测青海高原地区新生儿高胆红素血症的准确性欠佳，本研究基于 c.211G>A 位点变异型频率及 A 等位基因频率、TBil、IDB、AST、ALB 构建青海高原地区新生儿高胆红素血症风险列线图模型，结果显示 C-index 为 0.889（95%CI：0.807~0.938），可见多指标联合预测模型的效能较高，后续可进一步比较不同模型预测不同地区新生儿高胆红素血症的准确性。本研究肯定了基于 *UGT1A1* 基因多态性构建的青海高原地区新生儿高胆红素血症风险预测模型预测效能，但亦存在一定局限性，如纳入的危险因素种类较多，结果可能受选择偏倚的影响；纳入病例均来源于同一医院，尚需更多外部数据对本研究相关结论进行验证，尤其是不同种族或民族间的多中心队列研究。

综上所述，青海高原地区新生儿高胆红素血症 *UGT1A1* 基因最常见的变异位点为 c.211G>A，且基于 c.211G>A 位点变异型频率及 A 等位基因频率等中国高原地区新生儿高胆红素血症的影响因素构建的列线图模型预测效能较好。

参 考 文 献

[1] Qattea I, Farghaly MAA, Elgendy M, et al. Neonatal hyperbilirubinemia and bilirubin neurotoxicity in hospitalized neonates: Analysis of the US Database[J]. Pediatr Res, 2022, 91(7): 1662-1668.

[2] Khan A, Kim TY. Neonatal hyperbilirubinemia: Recommendations for diagnosis and management in the emergency department[J]. Pediatr Emerg Med Pract, 2022, 19(1): 1-24.

[3] Kuitunen I, Kiviranta P, Sankilampi U, et al. Ursodeoxycholic acid as adjuvant treatment to phototherapy for neonatal hyperbilirubinemia: A systematic review and meta-analysis[J]. World J Pediatr, 2022, 18(9): 589-597.

[4] Kuniyoshi Y, Tokutake H, Takahashi N, et al. Regional variation in the development of neonatal hyperbilirubinemia and relation with sunshine duration in Japan: An ecological study[J]. J Matern Fetal Neonatal Med, 2022, 35(25): 4946-4951.

[5] Fanello C, Lee SJ, Bancone G, et al. Prevalence and risk factors of neonatal hyperbilirubinemia in a semi-rural area of the democratic republic of Congo: A cohort study[J]. Am J Trop Med Hyg, 2023, 109(4): 965-974.

[6] Yang XJ, Weber AA, Mennillo E, et al. Oral arsenic administration to humanizedUDP-glucuronosyltransferase1 neonatal mice induces UGT1A1 through a dependence on Nrf2 and PXR[J]. J Biol Chem, 2023, 299(3): 102955.

[7] Horinouchi T, Maeyama K, Nagai M, et al. Genetic analysis of UGT1A1 polymorphisms using preserved dried umbilical cord for assessing the potential of neonatal jaundice as a risk factor for autism spectrum disorder in children[J]. J Autism Dev Disord, 2022, 52(2): 483-489.

[8] 中华医学会儿科学分会新生儿学组,《中华儿科杂志》编辑委员会. 新生儿高胆红素血症诊断和治疗专家共识[J]. 中华儿科杂志, 2014, 52(10): 745-748.

[9] Lazarus G, Francie J, Roeslani RD, et al. Role of ursodeoxycholic acid in neonatal indirect hyperbilirubinemia: A systematic review and meta-analysis of randomized controlled trials[J]. Ital J Pediatr, 2022, 48(1): 179.

[10] Walker-Pizarro N, Teran CG. Letter to the editor regarding the article: Probiotics for the management of neonatal hyperbilirubinemia: A systematic review of randomized controlled trial[J]. J Matern Fetal Neonatal Med, 2022, 35(25): 6915-6916.

[11] Hung YL, Chang PF, Huang CS. Molecular biology of glucose-6-phosphate dehydrogenase and UDP-glucuronosyltransferase 1A1 in the development of neonatal unconjugated hyperbilirubinemia[J]. Pediatr Neonatol, 2024, 65(5): 419-426.

[12] 谈钰培, 钟丹妮, 赵科, 等. 不明原因新生儿高胆红素血症与 *UGT1A1* 基因突变的关系[J]. 广西医科大学学报, 2022, 39(5): 809-814.

[13] Yanagi T, Nakahara S, Maruo Y. Bilirubin uridine diphosphate-glucuronosyltransferase polymorphism as a risk factor for prolonged hyperbilirubinemia in Japanese preterm infants[J]. J Pediatr, 2017, 190(1): 159-162.e1.

[14] Liu W, Chang LW, Xie M, et al. Correlation between UGT1A1 polymorphism and neonatal hyperbilirubinemia of neonates in Wuhan[J]. J Huazhong Univ Sci Technolog Med Sci, 2017, 37(5): 740-743.

[15] Raturi M, Adhikari B, Kalra A, et al. Reporting an immune-mediated neonatal hyperbilirubinemia following Rh (D) isoimmunization in an Indian term newborn bearing A_{MFA} B Rh (D) positive phenotype[J]. Indian J Pathol Microbiol, 2023, 66(3): 669-671.

[16] Pathak A, Siddalingesha R, Prasad KN, et al. To study the association between various levels of cord serum albumin (CSA) and significant neonatal hyperbilirubinemia requiring interventions like phototherapy or exchange transfusion[J]. J Family Med Prim Care, 2022, 11(6): 2483-2487.

[17] Juusela AL, Cordero L, Gimovsky M, et al. Correlation of bile acids and aspartate-aminotransferase with outcomes in cholestasis of pregnancy[J]. J Neonatal Perinatal Med, 2020, 13(4): 513-519.

[18] Chakrahari S, Patil M, Bijapure HR. Umbilical cord blood bilirubin, albumin, reticulocyte count, and nucleated red blood cells to predict subsequent hyperbilirubinemia in term neonates: A prospective observational study[J]. Cureus, 2023, 15(4): e37598.

[19] 石羽, 曹娟, 王红怡, 等. 高胆红素血症新生儿血清 miR-122 水平与肝功能各项指标及葡萄糖-6-磷酸脱氢酶缺乏的相关性研究[J]. 中西医结合肝病杂志, 2022, 32(1): 23-27.

[20] Zhou TC, Li X, Li H, et al. Concurrence of novel mutations causing Gilbert's and Dubin-Johnson syndrome with poor clinical outcomes in a Han Chinese family[J]. J Hum Genet, 2023, 68(1): 17-23.

收稿日期：2024-10-25

中国知网 https://www.cnki.net
